# SUMO2/3 conjugation of TDP-43 protects against aggregation

**DOI:** 10.1126/sciadv.adq2475

**Published:** 2025-02-21

**Authors:** Enza Maria Verde, Francesco Antoniani, Laura Mediani, Valentina Secco, Samuele Crotti, Maria Celidea Ferrara, Jonathan Vinet, Aleksandra Sergeeva, Xiao Yan, Carsten Hoege, Cristiana Stuani, Francesca Paron, Tzu-Ting Kao, Rohit Shrivastava, Jolanta Polanowska, Aymeric Bailly, Alessandro Rosa, Eleonora Aronica, Anand Goswami, Neil Shneider, Anthony A. Hyman, Emanuele Buratti, Dimitris Xirodimas, Titus M. Franzmann, Simon Alberti, Serena Carra

**Affiliations:** ^1^ Department of Biomedical, Metabolic and Neural Sciences, University of Modena and Reggio Emilia, Modena 41125, Italy.; ^2^ Centro Interdipartimentale Grandi Strumenti (CIGS), University of Modena and Reggio Emilia, Modena 41125, Italy.; ^3^ Center for Molecular and Cellular Bioengineering, Biotechnology Center, Technische Universität Dresden, Dresden 01307, Germany.; ^4^ Max Planck Institute of Molecular Cell Biology and Genetics, Dresden 01307, Germany.; ^5^ Molecular Pathology Lab, International Centre for Genetic Engineering and Biotechnology (ICGEB), Trieste 34149, Italy.; ^6^ Department of Neurology, Center for Motor Neuron Biology and Disease, Columbia University, New York, NY 10032, USA.; ^7^ Department of Neurology, Eleanor and Lou Gehrig ALS Center, Columbia University, New York, NY 10032, USA.; ^8^ CRBM, Université de Montpellier, CNRS, Montpellier Cedex 05, 34293, France.; ^9^ Department of Biology and Biotechnologies “Charles Darwin”, Sapienza University of Rome, Rome, Italy.; ^10^ Center for Life Nano- & Neuro-Science, Fondazione Istituto Italiano di Tecnologia (IIT), Rome, Italy.; ^11^ Amsterdam UMC, University of Amsterdam, Department of (Neuro)Pathology, Amsterdam Neuroscience, Amsterdam, Netherlands.

## Abstract

Cytosolic aggregation of the RNA binding protein TDP-43 (transactive response DNA-binding protein 43) is a hallmark of amyotrophic lateral sclerosis and frontotemporal dementia. Here, we report that during oxidative stress, TDP-43 becomes SUMO2/3-ylated by the SUMO E3 ligase protein PIAS4 (protein inhibitor of activated STAT 4) and enriches in cytoplasmic stress granules (SGs). Upon pharmacological inhibition of TDP-43 SUMO2/3-ylation or PIAS4 depletion, TDP-43 enrichment in SGs is accompanied by irreversible aggregation. In cells that are unable to assemble SGs, SUMO2/3-ylation of TDP-43 is strongly impaired, supporting the notion that SGs are compartments that promote TDP-43 SUMO2/3-ylation during oxidative stress. Binding of TDP-43 to UG-rich RNA antagonizes PIAS4-mediated SUMO2/3-ylation, while RNA dissociation promotes TDP-43 SUMO2/3-ylation. We conclude that SUMO2/3 protein conjugation is a cellular mechanism to stabilize cytosolic RNA-free TDP-43 against aggregation.

## Teaser

During oxidative stress, TDP-43 is SUMO2/3-ylated by the SUMO E3 ligase PIAS4 to prevent its aggregation inside stress granules.

## INTRODUCTION

Transactive response DNA-binding protein 43 (TDP-43) is a nuclear RNA binding protein (RBP) involved in RNA metabolism. Cytosolic inclusions of TDP-43 are a pathological hallmark of amyotrophic lateral sclerosis (ALS) and frontotemporal dementia (FTD) (*1*, *2*). TDP-43 has a high propensity to aggregate because of its low solubility in cells and in vitro (*2*). The aggregation propensity of TDP-43 is increased by ALS/FTD-linked mutations and upon exposure to stress (*2*, *3*), and has been observed in patients with *C9orf72* hexanucleotide repeat expansion, the most common genetic cause of sporadic and familial ALS (fALS) and FTD (*4*). Because TDP-43 aggregation is central to familial and sporadic ALS/FTD, approaches aimed at preventing TDP-43 aggregation hold promise for future ALS/FTD treatments.

How cells control TDP-43 aggregation is poorly understood. Modifiers of TDP-43 solubility include molecular chaperones and posttranslational modifications (PTMs) (*2*, *5*). Besides ubiquitination, which is a key PTM required to clear aggregation-prone proteins, phosphorylation of TDP-43 is emerging as a protective response to counteract its misfolding. Phosphorylation of TDP-43 decreases its assembly into condensates and suppresses TDP-43 aggregation and toxicity (*6*).

TDP-43 can also be SUMOylated. SUMOylation involves the conjugation of a substrate protein with either SUMO1 or SUMO2/3, via mono-, multi-mono, or poly-SUMOylation (*7*). Under normal growth conditions, cells prefer to modify proteins with SUMO1 (referred to as SUMO1-ylation), while SUMO2 and SUMO3 are usually conjugated in the form of SUMO2/3 chains (referred to as SUMO2/3-ylation) during cellular stress (*8*). TDP-43 is SUMO1-ylated on a lysine residue at position 136 (K136), which retains it in the nucleus, preventing its aggregation in the cytoplasm (*9*) and affects its splicing activity (*10*). In addition, mass spectrometry studies identified several SUMO2 sites in TDP-43 (*11*–*13*). Yet, the role of TDP-43 SUMO2/3-ylation is currently unknown.

Stress conditions trigger the accumulation of TDP-43 in cytosolic stress granules (SGs), which are dynamic RNA-containing condensates (*14*). The role of SGs in modulating TDP-43 aggregation is ambiguous. TDP-43 can misfold and aggregate independently of SGs (*15*), but chronic induction of SGs has been shown to trigger the formation of cytosolic TDP-43 inclusions that cause neurotoxicity (*16*). Of note, SG assembly coincides with a marked increase in protein SUMO2/3-ylation (*17*). Advanced mass spectrometry–based proteomics revealed a strong overlap between the SUMO2/3 and the SG proteomes, including many aggregation-prone RBPs (*11*–*13*). Functionally, SUMO2/3-ylation has been shown to maintain SGs in a dynamic state: SUMOylation inhibition impairs SG disassembly, but the underlying mechanism is still unknown (*18*, *19*).

In this paper, we report that upon oxidative stress, TDP-43 is modified with SUMO2/3 chains. Using experiments in cells, we show that conjugation of TDP-43 with SUMO2/3 coincides with SG assembly. Pharmacological inhibition of TDP-43 SUMO2/3-ylation triggers TDP-43 aggregation inside SGs. Using a knockdown approach, we identify the SUMO E3 ligase protein inhibitor of activated STAT 4 (PIAS4) as the enzyme that modifies TDP-43 with SUMO2/3. Using a reconstitution system, we demonstrate that PIAS4-mediated SUMO2/3-ylation of TDP-43 is antagonized by UG-rich RNA, while the absence of RNA promotes TDP-43 SUMO2/3-ylation. Furthermore, PIAS4-mediated SUMO2/3-ylation increases the solubility of TDP-43 and prevents its aggregation in the cytoplasm. Of note, the cytoplasmic immunoreactivity of PIAS4 was decreased in motor neurons of the human spinal cord in fALS cases with TDP-43 and C9orf72 mutations. We conclude that modification with SUMO2/3 chains is a mechanism to maintain the solubility of RNA-free TDP-43 during stress.

## RESULTS

### Oxidative stress causes TDP-43 SUMO2/3-ylation and enrichment in cytoplasmic SGs

**Fig. 1.**
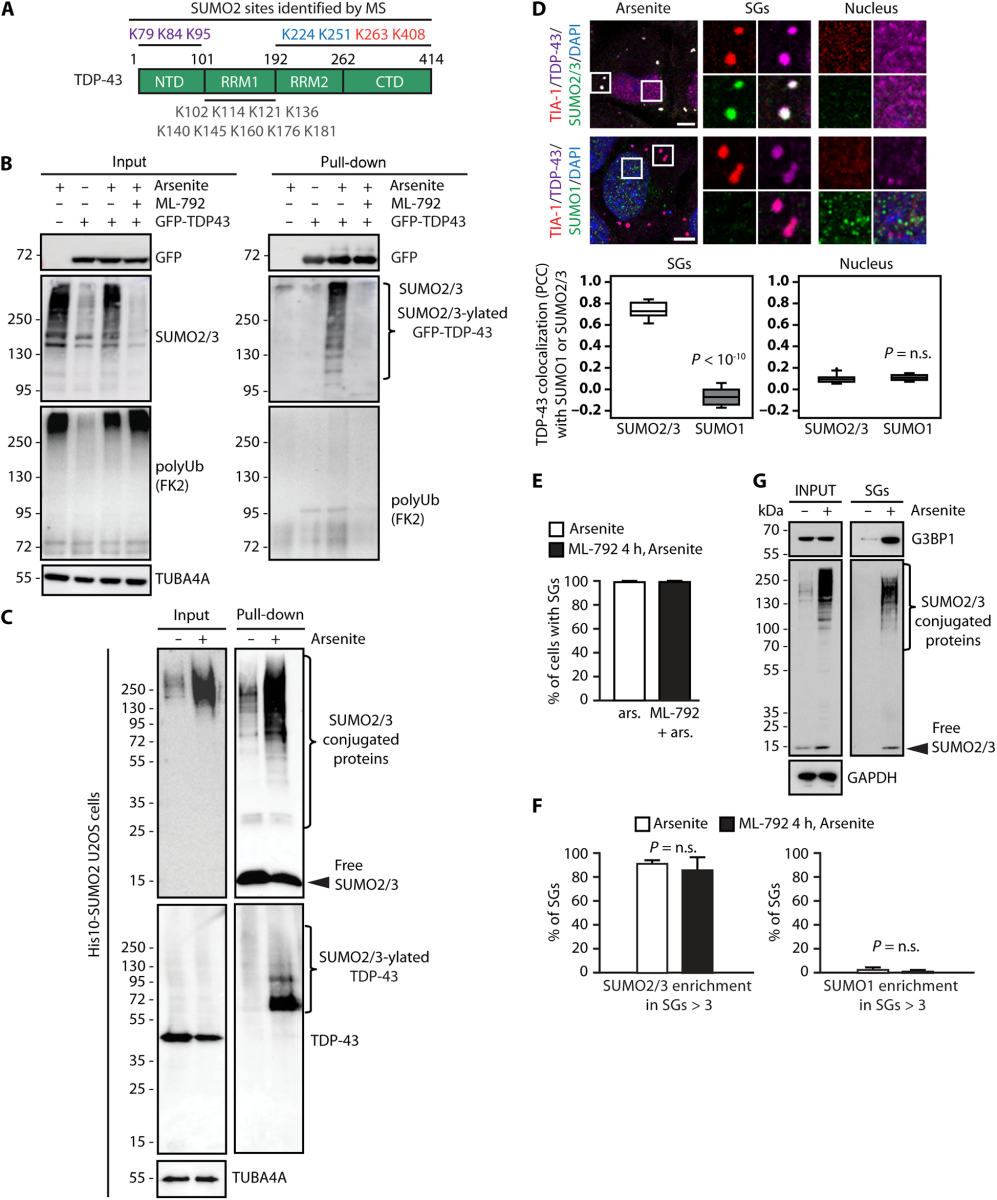
Upon oxidative stress, TDP-43 is SUMO2/3-ylated and colocalizes with SGs. (**A**) Schematic representation of TDP-43 protein domains and the SUMO2 sites identified by mass spectrometry (*13*). (**B**) Immunoprecipitation under denaturing conditions of GFP-TDP-43 from U2OS cells untreated or treated with arsenite (500 μM, 1 hour), where indicated cells were incubated with ML-792 (2 μM, 2 hours), before the addition of arsenite. Immunoblots of denatured total protein (input) and beads (pull-down) fractions are shown. TUBA4A was used as loading control. (**C**) Ni-NTA pull-down of His-SUMO2 under denaturing conditions from His10-SUMO2 U2OS cells left untreated or treated with arsenite (500 μM, 1 hour). Immunoblots of total SUMO2/3 proteins and endogenous TDP-43 are shown. TUBA4A was used as loading control. (**D**) Confocal imaging of TIA-1, TDP-43, SUMO2/3, SUMO1, and DAPI in arsenite-treated U2OS cells. Colocalization of TDP-43 and SUMO1 or SUMO2/3 inside SGs and nuclei is shown (Pearson’s correlation coefficient/PCC; mean ± SEM; SUMO1: *n* = 2946 SGs, *n* = 228 nuclei; SUMO2/3: *n* = 1964 SGs, *n* = 203 nuclei). Student’s *t* test. Scale bar, 5 μm. (**E**) Percentage of HeLa Kyoto cells forming SGs following arsenite treatment alone or preceded by ML-792 pretreatment (mean ± SEM, *n* = 3). Student’s *t* test. (**F**) HeLa Kyoto cells were treated as reported in (E), followed by confocal imaging. The enrichment of SUMO1 and SUMO2/3 inside SGs is shown (mean ± SEM, *n* = 3. Total number of SGs analyzed: 10,859 to 11,896 for SUMO1; 11,345 to 12,296 for SUMO2/3). Fluorescence intensity >3 corresponds to SGs highly enriched for SUMO1 and SUMO2/3. One-way ANOVA, followed by Bonferroni-Holm post hoc test. (**G**) Immunoblots of total and SG-enriched fractions from His6-SUMO2-HEK293T cells untreated or treated with arsenite (500 μM, 1 hour). GAPDH was used as loading control.

Mass spectrometry studies identified several SUMO2 sites in TDP-43, which are distributed throughout the protein sequence, the N terminus, the two RNA recognition motifs (RRM1 and RRM2), and the C terminus (Fig. 1A) (*12*, *13*). To test for TDP-43 SUMO2/3-ylation in response to oxidative stress, we overexpressed GFP-tagged TDP-43 in U2OS cells and immunoprecipitated it under denaturing conditions to remove noncovalent interactions and prevent deconjugation. Cells were either left untreated or exposed to the oxidative agent sodium arsenite. Arsenite treatment induced global protein SUMO2/3-ylation, which was largely prevented by preincubation with the specific SUMO E1 activating enzyme (SAE1/SAE2) inhibitor ML-792 (Fig. 1B, input). GFP-TDP-43 was one of the proteins that was SUMO2/3-ylated following arsenite stress. SUMO2/3-ylated TDP-43 was visible as a high–molecular weight smear on immunoblots, and this smear was reduced upon the addition of ML-792 (Fig. 1B, pull-down). By contrast, arsenite treatment did not induce SUMO1 levels, nor conjugation of GFP-TDP-43 with SUMO1 (fig. S1A). Proteotoxic stress conditions, such as heat shock or proteasome inhibition, have been shown to increase the overall levels of polyubiquitination and conjugation of proteins with mixed polyubiquitin-SUMO2/3 chains (*18*, *20*). We thus tested whether GFP-TDP-43 becomes polyubiquitinated upon oxidative stress. The overall level of polyubiquitinated proteins was increased upon arsenite treatment (Fig. 1B, input), but polyubiquitination of GFP-TDP-43 was barely detectable (Fig. 1B, pull-down). Thus, upon oxidative stress, GFP-TDP-43 is preferentially conjugated to SUMO2/3 chains rather than with mixed polyubiquitin-SUMO2/3 chains. Next, to exclude the possibility that the GFP-tag might influence TDP-43 SUMO2/3-ylation, we used FLAG-tagged TDP-43. Immunoprecipitation under denaturing conditions showed that FLAG-TDP-43 is conjugated to SUMO2/3 in response to arsenite treatment (fig. S1B, pull-down). We further confirmed that endogenous TDP-43 is conjugated to SUMO2/3 following arsenite treatment using U2OS cells that stably express His10-SUMO2 (Fig. 1C). On the basis of the molecular weight of endogenous TDP-43 in the pull-down fraction, we cannot exclude the possibility that besides polySUMO2/3-ylation, a fraction of TDP-43 could be mono and/or multi-monoSUMO2-ylated (Fig. 1C). The subcellular localization of SUMOylated proteins can be influenced by their interaction with proteins that contain SUMO-interacting motifs (SIMs), including the SUMO enzymes, which are enriched in the cell nucleus (*21*). For example, SUMO1-ylation of TDP-43 at K136 promotes its nuclear retention (*10*). We next investigated whether arsenite-induced TDP-43 SUMO2/3-ylation affects TDP-43 subcellular localization. In untreated U2OS cells, TDP-43 was predominantly nuclear, as was SUMO2/3. However, upon arsenite treatment, a fraction of TDP-43 and SUMO2/3 translocated into the cytoplasm and localized to cytoplasmic SGs (fig. S1C, left). By contrast, SUMO1 was predominantly localized in nuclear foci reminiscent of PML nuclear bodies, which are enriched for SUMO1-conjugated PML (*21*), but was not detected inside SGs (fig. S1C, right). Quantification of confocal fluorescence microscopy images revealed colocalization between endogenous TDP-43 and SUMO2/3, but not with SUMO1, inside TIA-1–positive SGs (Fig. 1D). No colocalization was observed between nuclear TDP-43 and nuclear SUMO1 or SUMO2/3 (Fig. 1D) within the same cells, indicating specificity. Together, our data demonstrate that upon oxidative stress, (i) TDP-43 becomes SUMO2/3-ylated, and (ii) both TDP-43 and SUMO2/3 enrich in SGs.

### Conjugated and free SUMO2/3 localize to SGs

Our data indicate that SUMO2/3 accumulates in SGs upon oxidative stress. However, previous reports did not detect SUMO2/3 inside SGs. We set out to investigate how the different observations may be explained. We noticed that previous studies used different antibodies and formaldehyde, rather than methanol to fix and stain cells (*18*, *22*). Recently, formaldehyde fixation was shown to both enhance and diminish the detection of proteins inside condensates (*23*). In agreement with previous reports (*18*, *22*), SUMO2/3 was not detected in SGs using formaldehyde fixation. However, SUMO2/3 was detected in SGs with methanol as fixative (fig. S1D). No signal was observed when cells were incubated only with secondary antibodies (fig. S1D), demonstrating that the SUMO2/3 signal was specific. Besides U2OS cells (Fig. 1D) and HeLa cells (fig. S1D), we confirmed the localization of SUMO2/3 to arsenite-induced SGs also in neuronal SH-SY5Y cells (fig. S1E).

We used three additional approaches to verify the presence of both free and conjugated SUMO2/3 inside SGs. First, we overexpressed YFP-tagged SUMO2 [wild type (WT)] and the nonconjugatable SUMO2 (dGG) variant in HeLa cells (fig. S1F) (*24*). Upon arsenite treatment, a fraction of both YFP-SUMO2-WT and SUMO2-dGG enriched in SGs (fig. S1G). By contrast, YFP-SUMO2-WT and YFP-SUMO2-dGG did not colocalize with nuclear SC35 splicing speckles (fig. S1H), another type of condensate (*25*), demonstrating specificity. Inhibition of SUMOylation with ML-792 did not affect SG assembly (Fig. 1E), and it did not decrease SUMO2/3 enrichment inside SGs (Fig. 1F), indicating that free SUMO2/3 can enrich in SGs. By contrast, we observed no enrichment of SUMO1 inside SGs, regardless of the pretreatment with ML-792 (Fig. 1F). Second, we used Flag-Affimers that selectively interact with free and conjugated SUMO2 (FLAG-S2B3) or SUMO1 and SUMO2 (FLAG-S1S2D5) (*26*). Flag-Affimers colocalized with arsenite-induced SGs (fig. S1, I and J). Third, using established protocols (*27*), we prepared SG-enriched protein fractions from cells treated with arsenite. The SG-enriched fractions contained G3BP1, and free and conjugated SUMO2/3 (Fig. 1G). We conclude that SUMO2/3 partitions into SGs and that SGs contain both unconjugated and conjugated SUMO2/3.

### SUMO2/3-ylation maintains TDP-43 mobility inside SGs

**Fig. 2.**
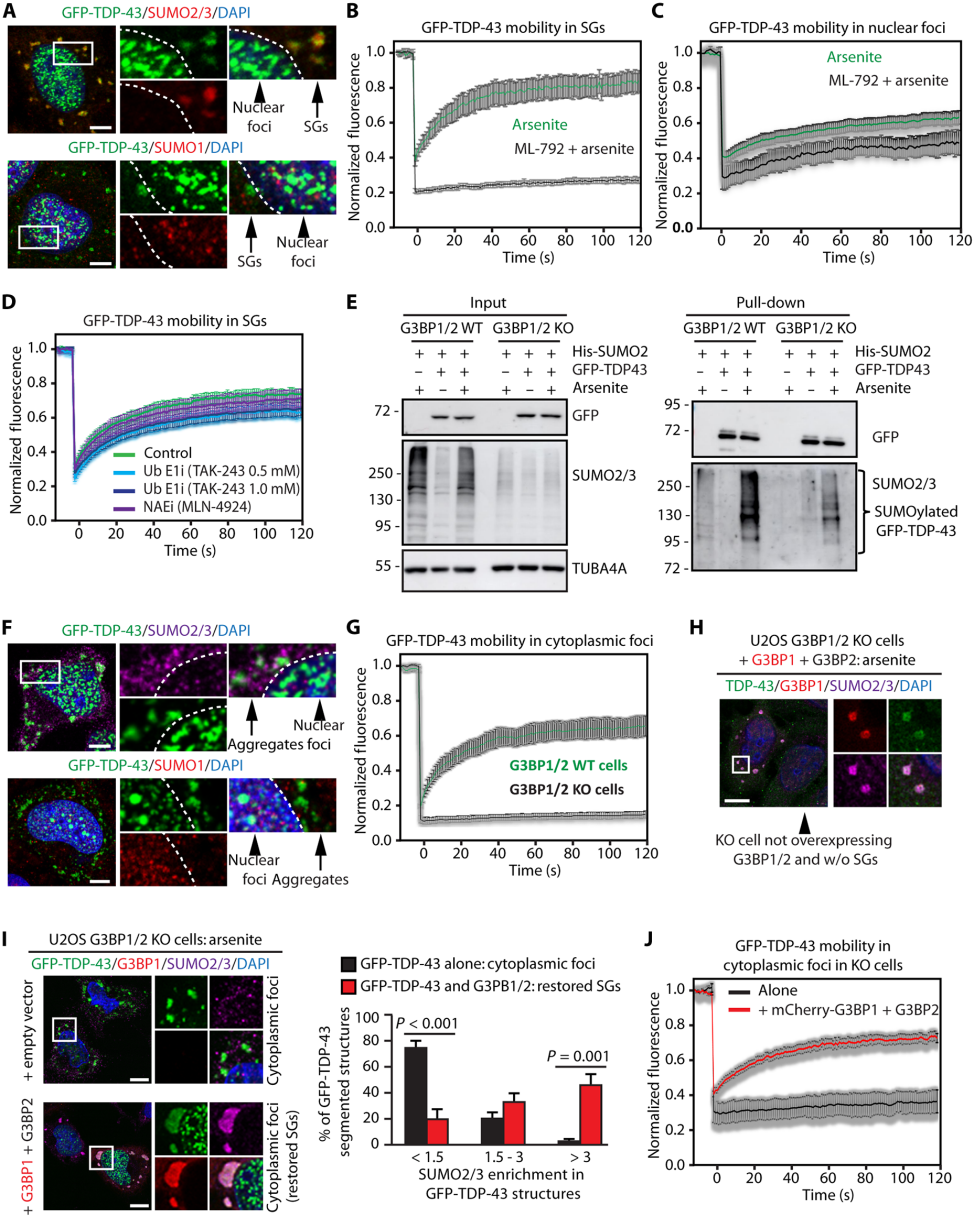
SUMO2/3-ylation maintains TDP-43 mobility inside SGs. (**A**) Confocal imaging of GFP-TDP-43, SUMO2/3, SUMO1, and DAPI in arsenite-treated U2OS cells. Scale bars, 5 μm. (**B**) FRAP curves of GFP-TDP-43 inside SGs in U2OS cells treated with arsenite alone or pretreated with ML-792 (mean ± SEM, *n* = 11 and 15). (**C**) FRAP curves of GFP-TDP-43 inside nuclear foci in U2OS cells treated with arsenite alone or pretreated with ML-792 (mean ± SEM, *n* = 15 and 21). (**D**) FRAP curves of GFP-TDP43 inside SGs in U2OS cells during arsenite stress with a pretreatment with either Ub E1i/TAK-243 or NAEi1/MLN-4924 (mean ± SEM, *n* = 12, control, or Ub E1i 0.5 μM, or 1.0 μM; *n* = 9, NAEi). (**E**) Immunoprecipitation under denaturing conditions of GFP-TDP-43 from U2OS G3BP1/2 WT and KO cells untreated or arsenite treated (500 μM, 1 hour). Immunoblots of denatured total protein (input) and beads (pull-down) fractions are shown. TUBA4A was used as loading control. (**F**) Confocal imaging of GFP-TDP-43, SUMO2/3, SUMO1, and DAPI in arsenite-treated U2OS G3BP1/2 KO cells. Scale bars, 5 μm. (**G**) FRAP curves of GFP-TDP-43 inside cytoplasmic foci in U2OS G3BP1/2 WT and KO cells treated with arsenite (mean ± SEM, *n* = 6 and 14, respectively). (**H**) Confocal imaging of overexpressed mCherry-G3BP1, endogenous TDP-43, SUMO2/3, and DAPI in U2OS G3BP1/2 KO arsenite-treated cells overexpressing mCherry-G3BP1 and G3BP2-myc-DDK (+ G3BP1 + G3BP2). The arrow indicates a nontransfected cell lacking SGs. Scale bar, 10 μm. (**I**) Confocal imaging of GFP-TDP-43, mCherry-G3BP1, SUMO2/3, and DAPI in U2OS G3BP1/2 KO cells overexpressing an empty vector or mCherry-G3BP1 and G3BP2-myc-DDK (+ G3BP1 + G3BP2) and arsenite treated. Quantification of SUMO2/3 enrichment inside GFP-TDP-43 aggregates and SGs (mean ± SEM, *n* = 4). One-way ANOVA, followed by Bonferroni-Holm post hoc test. Scale bars, 10 μm. (**J**) FRAP curves of GFP-TDP-43 inside cytoplasmic foci in U2OS G3BP1/2 KO cells transfected as described in (I) (mean ± SEM, *n* = 4 and 15).

Considering the conjugation of SUMO2/3 to TDP-43 upon oxidative stress and its colocalization with SUMO2/3 inside SGs, we then tested whether SUMO2/3-ylation influences the mobility of TDP-43 inside SGs. To investigate TDP-43 mobility, we quantified the dynamics of TDP-43 inside SGs using fluorescence recovery after photobleaching (FRAP). To this end, we compared the mobility of TDP-43 in two different condensates, both of which are enriched for TDP-43 upon arsenite treatment: nuclear condensates (*28*), which exhibit poor colocalization with SUMO2/3 (Fig. 1D), and cytoplasmic SGs, which strongly colocalized with SUMO2/3, but not with SUMO1 (Fig. 2A). GFP-TDP-43 was mobile inside arsenite-induced SGs (Fig. 2B, green curve). SUMO2/3-ylation inhibition with ML-792 did not prevent the recruitment of GFP-TDP-43 to SGs, but remarkably led to its immobilization (Fig. 2B, black curve). By contrast, GFP-TDP-43 was mobile in nuclear foci (Fig. 2C, green curve), and mobility was only mildly decreased upon ML-792 treatment (Fig. 2C, black curve). For comparison, we determined the mobility of mCherry-G3BP1 inside SGs. In contrast to TDP-43, G3BP1 exhibited high mobility inside SGs and its mobility was unchanged upon inhibition of SUMOylation (fig. S2A, red curve).

Concerning overall SG dynamics, in agreement with previous reports (*18*, *19*), inhibition of SUMOylation delayed the kinetics of SG disassembly (fig. S2B). Besides SUMOylation of other PTMs, such as ubiquitination and NEDDylation, can occur; inhibition of each of these PTMs was shown to affect SG dynamics (*22*, *29*). We thus investigated whether inhibition of ubiquitination or NEDDylation can also impair TDP-43 mobility inside SGs. To this end, we inhibited the ubiquitin-activating enzyme (Ub E1i) or the NEDD8-activating enzyme (NAEi) and probed for ubiquitination and NEDDylation, respectively. Both ubiquitination and NEDDylation levels were selectively reduced upon inhibition; however, the level of SUMO2/3-ylation was not altered (fig. S2C). We then pretreated U2OS cells with either Ub E1i or NAEi, followed by the addition of arsenite and analysis of GFP-TDP-43 mobility by FRAP. GFP-TDP-43 mobility inside SGs was not affected upon inhibition of NAE and only mildly reduced upon inhibition of Ub E1 (Fig. 2D). Ub E1i and NAEi did not influence the mobility of mCherry-G3BP1 inside SGs (fig. S2D), in agreement with previous data (*30*). Together, these results indicate that following arsenite treatment, cells specifically conjugate TDP-43 with SUMO2/3 chains to maintain its solubility and mobility inside SGs. Inhibition of SUMOylation coincides with a delay in SG disassembly kinetics (*18*, *19*) and leads to reduced mobility of TDP-43 inside SGs.

To provide further evidence for the interrelationship between TDP-43 SUMO2/3-ylation and its enrichment in cytoplasmic SGs, we used U2OS cells lacking two key proteins required for SG assembly, G3BP1 and G3BP2 [*G3BP1/2* knockout (KO)] (*31*). The SG marker TIA-1 was homogeneously distributed in G3BP1/2 KO cells treated with arsenite (fig. S2E), indicating the absence of SG formation in these cells. We then measured global protein SUMO2/3-ylation by immunoblotting. G3BP1/2 KO cells failed to induce protein SUMO2/3-ylation upon arsenite treatment (fig. S2F). By contrast, SUMO1 levels were similar in G3BP1/2 WT and KO cells untreated, exposed to arsenite stress during the acute stress phase, as well as during the stress recovery phase (fig. S2F). Of note, the SUMO2/3-ylation of GFP-TDP-43 was strongly decreased in G3BP1/2 KO cells compared to WT cells (Fig. 2E). We next used the proximity ligation assays (PLAs) to compare TDP-43 SUMO2/3-ylation in G3BP1/2 WT and KO cells. PLA was previously used to study PTMs such as phosphorylation, ubiquitination, and SUMOylation, and it can detect both protein SUMOylation and interaction of a protein of interest with SUMOylated proteins (*32*). Proximity between SUMO2/3 and TDP-43, as determined by the average number of PLA foci/cell, increased in WT cells treated with arsenite as compared with untreated cells (fig. S2G, white columns). The average number of PLA foci/cell was substantially reduced in KO cells compared to WT cells (fig. S2G, compare white and black columns); in addition, the SUMO2/3-TDP-43 PLA foci were mainly localized to the cytoplasm in both cell lines (fig. S2G). Together, these data indicate a tight correlation between the ability of cells to assemble cytoplasmic SGs and the efficiency of TDP-43 SUMO2/3-ylation.

Next, we investigated the localization of GFP-TDP-43 in G3BP1/2 KO cells using confocal microscopy analysis upon arsenite stress. GFP-TDP-43 formed both nuclear and cytoplasmic foci reminiscent of aggregates, which did not colocalize with SUMO2/3 and SUMO1 (Fig. 2F and fig. S2H), but instead colocalized with ubiquitin and the chaperone HSPA1A (fig. S2I). GFP-TDP-43 was immobile inside these cytoplasmic aggregates (Fig. 2G, black curve); this is in sharp contrast to the high mobility of GFP-TDP-43 inside SGs in WT cells (Fig. 2, B and G, green curves), which are enriched for SUMO2/3 (fig. S2H). In addition, the mobility of GFP-TDP-43 inside nuclear foci was similar in G3BP1/2 WT and KO cells treated with arsenite (fig. S2J), which were not enriched for SUMO2/3 in both cell lines (fig. S2H). We conclude that TDP-43 assembles into nondynamic aggregates in the cytoplasm of cells that are unable to form SGs.

To provide further evidence for the role of cytoplasmic SGs in mediating SUMO2/3-ylation and maintaining GFP-TDP-43 mobility, we restored SG assembly in G3BP1/2 KO cells by reexpressing mCherry-G3BP1 and G3BP2 (*33*). Expression of G3BP1 and G3BP2 enabled the formation of arsenite-induced SGs, which recruited both endogenous TDP-43 and SUMO2/3 (Fig. 2H, compare with the nontransfected cell). Expression of G3BP1 and G3BP2 rescued not only SG assembly, but also the enrichment of both GFP-TDP-43 and SUMO2/3 within SGs (Fig. 2I), and the TDP-43 mobility within SGs, as measured by FRAP (Fig. 2J, red curve).

In summary, these data indicate that SUMO2/3-ylation occurs more efficiently in cells that can assemble SGs and conjugation of SUMO2/3 to TDP-43 protects it from aggregation upon oxidative stress conditions in the cytoplasm.

### SUMO2/3-ylation regulates TDP-43 solubility upon oxidative stress

**Fig. 3.**
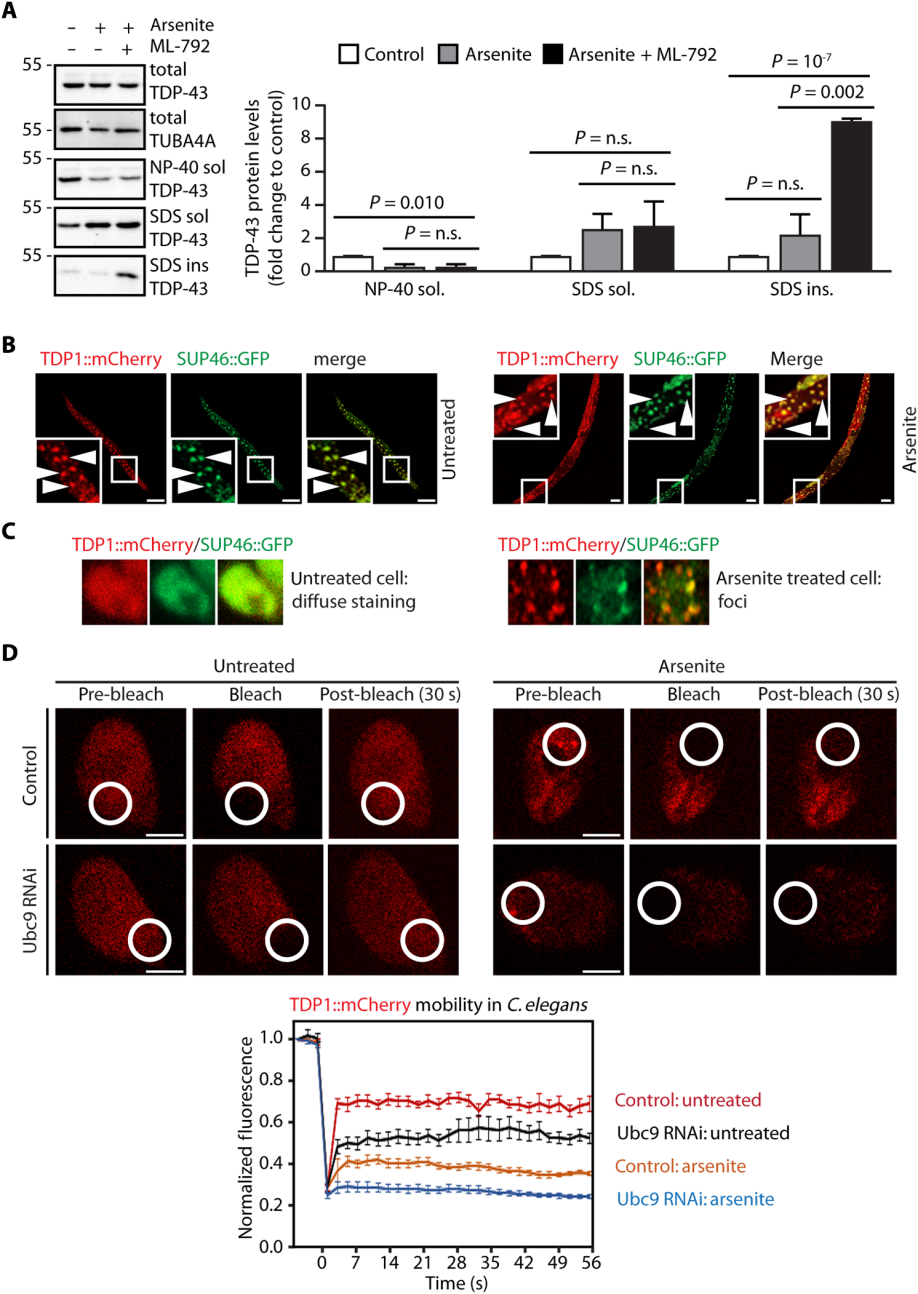
TDP-43 aggregates upon SUMO2/3-ylation inhibition. (**A**) Sequential fractionation of NP-40 soluble, SDS-soluble, and insoluble proteins from U2OS cells untreated or treated with arsenite with or without a pretreatment with ML-792 and quantification of endogenous TDP-43 protein levels, expressed as fold change compared to the control condition (mean ± SEM, *n* = 4). TUBA4A was used as loading control. One-way ANOVA, followed by Bonferroni-Holm post hoc test. (**B**) Confocal imaging of *C. elegans* overexpressing TDP1-mCherry and SUP-46-GFP untreated or arsenite treated. White arrowheads indicate somatic cells. Scale bars, 5 μm. (**C**) High magnification of one representative cell coexpressing TDP1-mCherry and SUP-46-GFP in untreated (left) or arsenite-treated (right) animals. (**D**) Representative images of FRAP experiments in TDP1-mCherry in Ubc9-proficient and -deficient (Ubc9 RNAi fed) worms untreated or arsenite treated. The bleached area is shown. Scale bars, 5 μm. FRAP curves of TDP1-mCherry in Ubc9-proficient and -deficient (Ubc9 RNAi fed) worms untreated or arsenite treated (mean ± SEM, *n* = 8 to 9).

We compared the subcellular localization of endogenous TDP-43 in U2OS G3BP1/2 WT and KO cells treated with arsenite (figs. S1C and S2E). A fraction of endogenous TDP-43 was recruited inside SGs in the G3BP1/2 WT cells exposed to sodium arsenite, while we noticed the formation of cytoplasmic foci reminiscent of protein aggregates in the arsenite-treated G3BP1/2 KO cells (fig. S3A). These cytoplasmic foci were largely devoid of SUMO2/3 (fig. S3, A to C), similar to the cytoplasmic aggregates formed by overexpressed GFP-TDP-43 in U2OS G3BP1/2 KO cells exposed to oxidative stress (Fig. 2F). To assess whether SUMO2/3-ylation regulates the solubility of endogenous TDP-43 during stress, we sequentially fractionated proteins based on their solubility in mild detergent (NP-40) versus strong denaturant (SDS), obtaining a fraction of NP-40 soluble proteins (fig. S3D, left) and, subsequently, a fraction of SDS-soluble proteins (fig. S3D, middle). We then centrifuged the fraction of SDS-soluble proteins to separate the SDS-insoluble proteins, which correspond to SDS-resistant aggregates (fig. S3D, right). The latter were then resolubilized with formic acid, using a previously established method (*34*). U2OS cells were either untreated or treated with arsenite, alone or combined with ML-792 to inhibit SUMOylation. In untreated cells, the majority of TDP-43 protein was found in the mild-detergent fraction. Upon arsenite treatment, TDP-43 was mainly found in the SDS-soluble fraction (Fig. 3A), suggesting reduced solubility. Upon inhibition of SUMOylation, TDP-43 accumulated in the SDS-insoluble fraction (Fig. 3A), suggesting that TDP-43 forms protein aggregates under these conditions. Of note, the total TDP-43 protein levels were similar in all conditions, indicating that there was no major degradation (Fig. 3A). We conclude that SUMO2/3-ylation is required to maintain TDP-43 in a soluble state during oxidative stress.

To test whether SUMOylation also regulates TDP-43 mobility in an animal model, we performed experiments in *Caenorhabditis elegans*. We chose a strain overexpressing mCherry-tagged TDP-1, the *C. elegans* ortholog of human TDP-43 and GFP-tagged SUP-46, an RBP that is recruited inside SGs (SUP46-GFP; TDP1-mCherry) (*35*). In somatic cells, TDP1-mCherry and SUP46-GFP were homogeneously distributed in the untreated animals (Fig. 3B, arrowheads, and Fig. 3C, left). Upon arsenite treatment, cells formed cytoplasmic foci containing both SUP46-GFP and TDP1-mCherry (Fig. 3B, arrowheads, and Fig. 3C, right). We then measured the mobility of TDP1-mCherry by FRAP in cells of live animals. In untreated conditions, TDP1-mCherry was highly mobile (Fig. 3D and fig. S3E). Upon treatment with increasing concentrations of arsenite, which led to the appearance of bright foci, we observed a dose-dependent decrease of TDP1-mCherry mobility (fig. S3E). To assess the role of SUMO in TDP1 mobility, we next silenced the SUMO-conjugating enzyme Ubc9 by RNAi feeding to inhibit SUMOylation. Silencing Ubc9 decreased the mobility of TDP1-mCherry in the cells of living animals even in the absence of external stressors and led to the complete immobilization of TDP1-mCherry upon treatment with a low dose of sodium arsenite (Fig. 3D). In combination with the experiments in human cells, the data indicate a conserved role for SUMO in protecting TDP-43 from aggregation upon stress.

### Lysine-to-arginine substitution affects TDP-43 mobility inside SGs

**Fig. 4.**
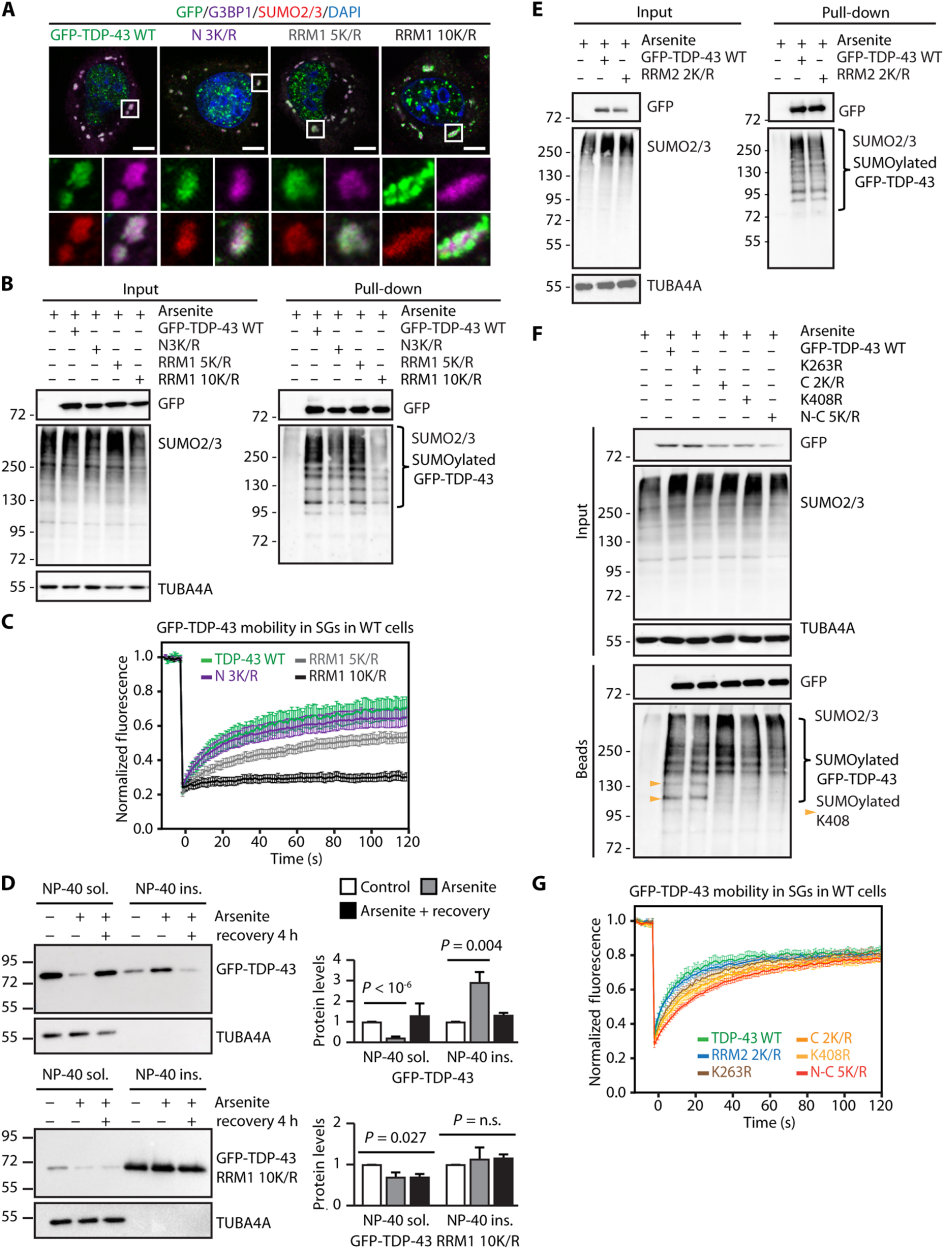
Impact of K/R substitution on TDP-43 mobility and solubility upon oxidative stress. (**A**) Confocal imaging of GFP-TDP-43 WT and the K/R variants, G3BP1, SUMO2/3, and DAPI in arsenite-treated U2OS cells. Scale bars, 5 μm. (**B**) Immunoprecipitation under denaturing conditions of GFP-TDP-43 WT and the K/R variants from U2OS cells treated with arsenite (500 μM, 1 hour). Immunoblots of denatured total protein (input) and beads (pull-down) fractions are shown. TUBA4A was used as loading control. (**C**) FRAP curves of GFP-TDP-43 WT and the K/R variants inside SGs in U2OS cells treated with arsenite (mean ± SEM, *n* = 9, WT; *n* = 12, N 3K/R; *n* = 16, RRM1 5K/R; *n* = 17, RRM1 10K/R). (**D**) Sequential fractionation of NP-40 soluble and insoluble proteins from U2OS cells overexpressing GFP-TDP-43 WT and RRM1 10K/R left untreated or treated with arsenite (500 μM, 1 hour). Where indicated, cells were allowed to recover for 4 hours following arsenite exposure (recovery); mean ± SEM, GFP-TDP-43 WT: *n* = 4, GFP-TDP-43 RRM1 10K/R: *n* = 3. One-way ANOVA, followed by Bonferroni-Holm post hoc test. TUBA4A was used as loading control. (**E** and **F**) Immunoprecipitation under denaturing conditions of GFP-TDP-43 WT and the RRM2 2K/R variant or the N- and C-terminal variants from U2OS cells treated with arsenite (500 μM, 1 hour). Immunoblots of denatured total protein (input) and beads (pull-down) fractions are shown. TUBA4A was used as loading control. (**G**) FRAP curves of GFP-TDP-43 WT and the RRM2 2K/R variant or the N- and C-terminal variants inside SGs in U2OS cells treated with arsenite (mean ± SEM, *n* = 7, WT; *n* = 14, RRM2 2K/R; *n* = 15, K263R; *n* = 14, C 2K/R; *n* = 16, K408R; *n* = 20, N-C 5K/R).

Substitution of lysine residues with arginine residues (K/R) in a given protein is a widely used approach to study how various PTMs influence protein functionality. To investigate how conjugation of SUMO2/3 to TDP-43 affects its dynamic properties, we mutated the SUMO2 sites identified by mass spectrometry located in either the N terminus or the RRM1 domain (fig. S4A). All GFP-TDP-43 K/R variants were predominantly localized inside the nucleus in untreated cells (fig. S4B), although RRM1 10K/R could also form aggregate-like structures that did not colocalize with SUMO2/3 in the cytoplasm of untreated cells (fig. S4C). Upon exposure to sodium arsenite, all K/R variants were recruited inside SGs (Fig. 4A). We noticed that RRM1 10K/R formed puncta reminiscent of protein aggregates at the border of SGs; these RRM1 10K/R-positive puncta did not colocalize with SUMO2/3 (Fig. 4A). Pull-down experiments under denaturing conditions showed that, except for RRM1 10K/R, the K/R variants could still be efficiently SUMO2/3-ylated (Fig. 4B). Moreover, RRM1 10K/R was completely immobile inside SGs, while the RRM1 5K/R was characterized by a slightly decreased mobility compared to GFP-TDP-43 WT (Fig. 4C). These observations suggest that the lysine residues located within the RRM1 domain are important to maintain TDP-43 in a soluble state upon stress. To further test this idea, we performed a solubility assay. GFP-TDP-43 WT was predominantly found in the NP-40 detergent-soluble fraction in untreated cells and after the stress recovery phase, while it accumulated in the NP-40 detergent-insoluble fraction upon arsenite treatment (Fig. 4D). Instead, RRM1 10K/R was prevalently found in the NP-40 detergent-insoluble fraction under all conditions tested, including the stress recovery phase (Fig. 4D).

We then mutated the SUMO2 sites identified by previous mass spectrometry studies (*12*, *13*) that are located within the RRM2 or the C terminus of TDP-43, as well as the SUMO2 sites located in both the N and C termini, generating a mutant referred to as N-C 5K/R (Fig. 1A and fig. S4D). All these variants were localized in the nucleus of untreated cells, and after arsenite treatment, they were all recruited inside SGs, with a homogeneous distribution within the SGs (fig. S4E, F). Concerning conjugation with SUMO2/3 upon arsenite treatment, we did not observe major differences when comparing these additional K/R variants to GFP-TDP-43 WT (Fig. 4, E and F), except for two SUMO2-conjugated bands that disappeared when the K408 residue was mutated into arginine (Fig. 4F, lanes 4 to 6 and yellow arrowheads). We then measured the mobility of these K/R variants inside arsenite-induced SGs by FRAP. The fluorescence recovery of the RRM2 2K/R variant was similar to that of the WT protein, while the fluorescence recovery of the C terminus and N-C 5K/R was slightly delayed (Fig. 4G). These data suggest that the K408 residue moderately contributes to both protein SUMO2/3-ylation and protein mobility upon oxidative stress.

Given that the association of RRMs with RNA involves lysine residues that form a salt bridge to the phosphodiester backbone (*36*), we then verified whether K/R substitution affects the ability of TDP-43 to interact with pre-mRNAs, influencing their splicing. A well-characterized splicing target of TDP-43 is the *CFTR* pre-mRNA: TDP-43 binds via its RRM1 domain to the UG-repeat of the *CFTR* pre-mRNA, promoting exon 9 skipping (*37*, *38*). We thus overexpressed a minigene containing the *CFTR* exon 9 in WT cells, expressing endogenous levels of TDP-43; in parallel, we transiently knocked down endogenous TDP-43 by small interfering RNA (siRNA), followed by replacement (add-back) by overexpression of either siRNA-resistant TDP-43 WT or the K/R variants together with the *CFTR* minigene (*37*, *39*). We then measured by reverse transcription polymerase chain reaction (RT-PCR) the ratio of exon 9 inclusion/skipping. TDP-43 silencing decreased the ratio of exon 9 inclusion/skipping, which was rescued by overexpression of TDP-43 WT (fig. S4G). All the K/R variants analyzed did not change the *CFTR* exon 9 splicing profile, except for RRM1 10K/R (fig. S4G). These data suggest that the RRM1 10K/R variant is unable to bind to RNA and regulate its splicing.

### PIAS4 is required to maintain TDP-43 mobility inside SGs and prevent its aggregation

**Fig. 5.**
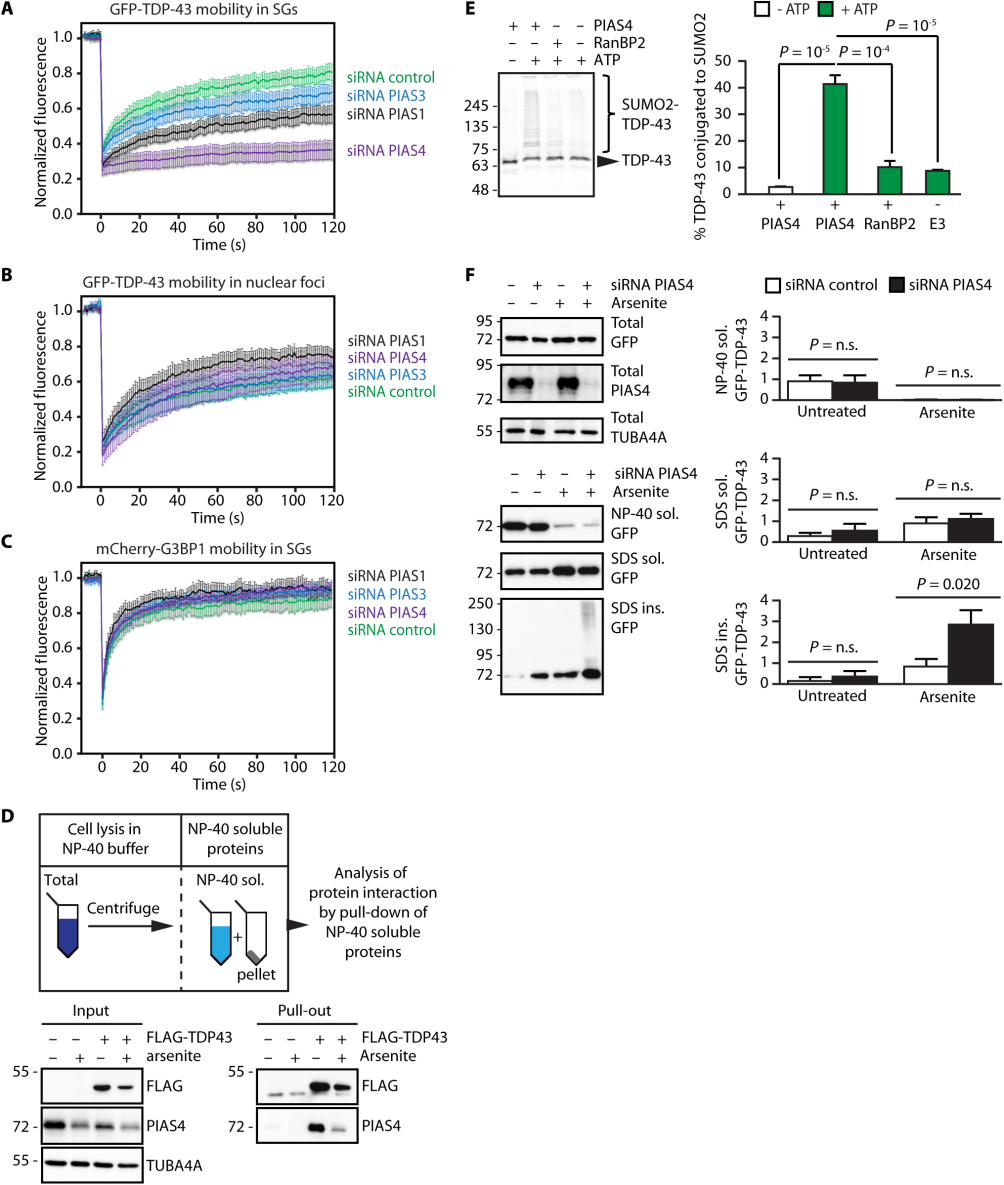
PIAS4 SUMO2-ylates TDP-43 and maintains TDP-43 solubility upon oxidative stress. (**A**) FRAP curves of GFP-TDP43 inside arsenite-induced SGs in U2OS cells transfected with the indicated siRNAs (mean ± SEM, *n* = 12, siRNA control; *n* = 12, siRNA PIAS1; *n* = 12, siRNA PIAS3; *n* = 16, siRNA PIAS4). (**B**) FRAP curves of GFP-TDP43 inside nuclear foci during arsenite stress in U2OS cells transfected with the indicated siRNAs (mean ± SEM, *n* = 9, siRNA control; *n* = 10, siRNA PIAS1; *n* = 6, siRNA PIAS3; *n* = 5, siRNA PIAS4). (**C**) FRAP curves of mCherry-G3BP1 inside arsenite-induced SGs in U2OS cells transfected with the indicated siRNAs (mean ± SEM, *n* = 6, siRNA control; *n* = 5, siRNA PIAS1; *n* = 7, siRNA PIAS3; *n* = 7, siRNA PIAS4). (**D**) Immunoprecipitation of FLAG-TDP-43 under nondenaturing conditions from U2OS cells untreated or arsenite treated (500 μM, 1 hour). Immunoblots of NP-40 soluble proteins (input) and beads (pull-down) fractions are shown. TUBA4A was used as loading control. (**E**) In vitro SUMO2-ylation of TDP-43-GFP in the absence or presence of either recombinant PIAS4, RanBP2, or ATP and quantification (mean ± SEM, *n* = 3). One-way ANOVA, followed by Bonferroni-Holm post hoc test. (**F**) Sequential fractionation of NP-40 soluble, SDS soluble and insoluble proteins from PIAS4-proficient and deficient U2OS cells left untreated or arsenite treated (500 μM, 1 hour) and quantification of GFP-TDP-43 protein levels (mean ± SEM, *n* = 4). TUBA4A was used as loading control. One-way ANOVA, followed by Bonferroni-Holm post hoc test.

Recent proteomic screens identified overlapping and specific sets of substrates for eight different SUMO E3 ligases (*40*, *41*). Two members of the PIAS family of proteins, PIAS1 and PIAS4, emerge as SUMO E3 ligases with TDP-43 as a preferred substrate. To test whether PIAS1 and PIAS4 influence TDP-43 mobility inside SGs during oxidative stress, we depleted PIAS1, PIAS3, and PIAS4 in U2OS cells with specific siRNA, which efficiently and selectively decreased the mRNA and protein levels of each targeted PIAS member (fig. S5, A and B). Depletion of the PIAS proteins did not exhibit any substantial influence on SG formation (fig. S5C). However, depletion of PIAS4 delayed SG disassembly, while depletion of PIAS1 and PIAS3 had only a moderate or no effect, respectively (fig. S5C). Depletion of PIAS4 coincided with the immobilization of GFP-TDP-43 inside SGs (Fig. 5A, purple curve). PIAS3 depletion mildly decreased GFP-TDP-43 mobility inside SGs, while PIAS1 depletion had a moderate effect (Fig. 5A, blue and black curves, respectively). The loss of mobility observed in PIAS4-depleted cells was specific to SGs, because the mobility of GFP-TDP-43 in nuclear foci was not affected and was even slightly higher compared to cells expressing an siRNA control (Fig. 5B). Moreover, the immobilization of TDP-43 upon PIAS4 depletion was not a general consequence of a less dynamic SG state, as mCherry-G3BP1 was mobile inside SGs under all conditions tested (Fig. 5C).

Next, we verified whether TDP-43 interacts with PIAS4 in cells. We overexpressed FLAG-tagged TDP-43, followed by immunoprecipitation under nondenaturing conditions, using the fraction of NP-40 soluble proteins. Endogenous PIAS4 interacted with FLAG-TDP-43 in both untreated and arsenite-treated U2OS cells (Fig. 5D). Of note, the decreased levels of both PIAS4 and TDP-43 in the input and pull-down fractions of arsenite-treated cells, compared to the untreated cells, are not due to reduced interaction, but rather to reduced solubility of TDP-43 in the NP-40 soluble fraction (see also Fig. 3A). To provide further evidence for the interaction between PIAS4 and TDP-43, we then used PLA to further study the proximity between these two proteins with spatial resolution in cells. We observed a significant PLA signal in untreated and arsenite-treated cells (fig. S5D). In untreated cells, the majority of the PIAS4-TDP-43 PLA foci were found in the cytoplasm, compared to the nucleus; following arsenite treatment, we observed a further increase in the % of PIAS4-TDP-43 PLA foci located in the cytoplasm, where SGs form (fig. S5D). We then quantified the enrichment of PIAS4 inside SGs by confocal microscopy. Although PIAS4 was mainly localized to the nucleus, we observed moderate levels of PIAS4 in more than 60% of SGs (fig. S5E).

To directly test whether PIAS4 SUMOylates TDP-43, we reconstituted the SUMOylation reaction in vitro following previously established protocols (*42*). The samples contained purified TDP-43-GFP, E1, E2 (Ubc9), and the PIAS4 E3 ligase, as well as SUMO2 and adenosine triphosphate (ATP). Because, even in the absence of an E3 ligase, many substrates can be SUMOylated in vitro (*43*), we carried out control reactions in the absence of ATP and/or E3 ligase. Besides PIAS4, we also used the E3 ligase RanBP2. Given the high molecular weight of RanBP2 (ca. 350 kDa), we used a functional fragment that harbors E3 catalytic activity (*44*) and we used its known target Sp100 as a positive control (*45*). Incubation with the RanBP2 fragment enhanced the SUMOylation of YFP-Sp100 (fig. S5F). Likewise, PIAS4 [both glutathione *S*-transferase (GST) tagged and untagged] was able to SUMOylate YFP-Sp100, yet to a lesser extent (fig. S5F), demonstrating that PIAS4 can act as a SUMO E3 ligase for the model substrate YFP-Sp100. Next, we investigated the SUMOylation of TDP-43-GFP. In the absence of ATP, but in the presence of PIAS4, only very low levels of conjugated SUMO2 were observed (Fig. 5E). In the presence of ATP and PIAS4, ~40% of TDP-43-GFP became SUMOylated (Fig. 5E). When the reaction was carried out either in the absence of PIAS4 or in the presence of RanBP2, the efficiency of SUMOylation dropped to ~10% (Fig. 5E). To exclude the possibility that the GFP protein itself might be conjugated to SUMO2, we used a truncated version of TDP-43 consisting of the N-terminal domain (NTD) fused to GFP. PIAS4 efficiently conjugated SUMO2 to full-length GFP-TDP-43, but not to NTD-GFP (fig. S5G). These data are in line with the observed SUMO2/3-ylation in cells of both GFP-TDP-43 and endogenous TDP-43 (Fig. 1, B and C). We conclude that PIAS4 can efficiently SUMOylate TDP-43 with SUMO2.

Given that inhibition of SUMOylation during arsenite treatment leads to the accumulation of TDP-43 in the SDS-insoluble fraction, we depleted PIAS4 in cells and determined its impact on TDP-43 solubility. Total expression levels of GFP-TDP-43 were similar in control and PIAS4-depleted cells (Fig. 5F, left). Arsenite treatment reduced GFP-TDP-43 solubility in mild detergent, regardless of whether PIAS4 was depleted or not, and TDP-43 accumulated in the SDS-soluble fraction (Fig. 5F, upper right). Arsenite caused the accumulation of GFP-TDP-43 in the SDS-insoluble fraction in both PIAS4-proficient and -deficient cells, yet TDP-43 aggregation was significantly stronger in PIAS4-depleted cells (Fig. 5F), similar to what was observed upon inhibition of SUMOylation with ML-792. Of note, silencing PIAS4 even led to GFP-TDP-43 accumulation in the SDS-insoluble fraction in the absence of stress, suggesting that PIAS4 may constantly survey RNA-free molecules of TDP-43 in cells (Fig. 5F).

### Binding to UG-rich RNAs competes with PIAS4-dependent TDP-43 SUMO2-ylation

**Fig. 6.**
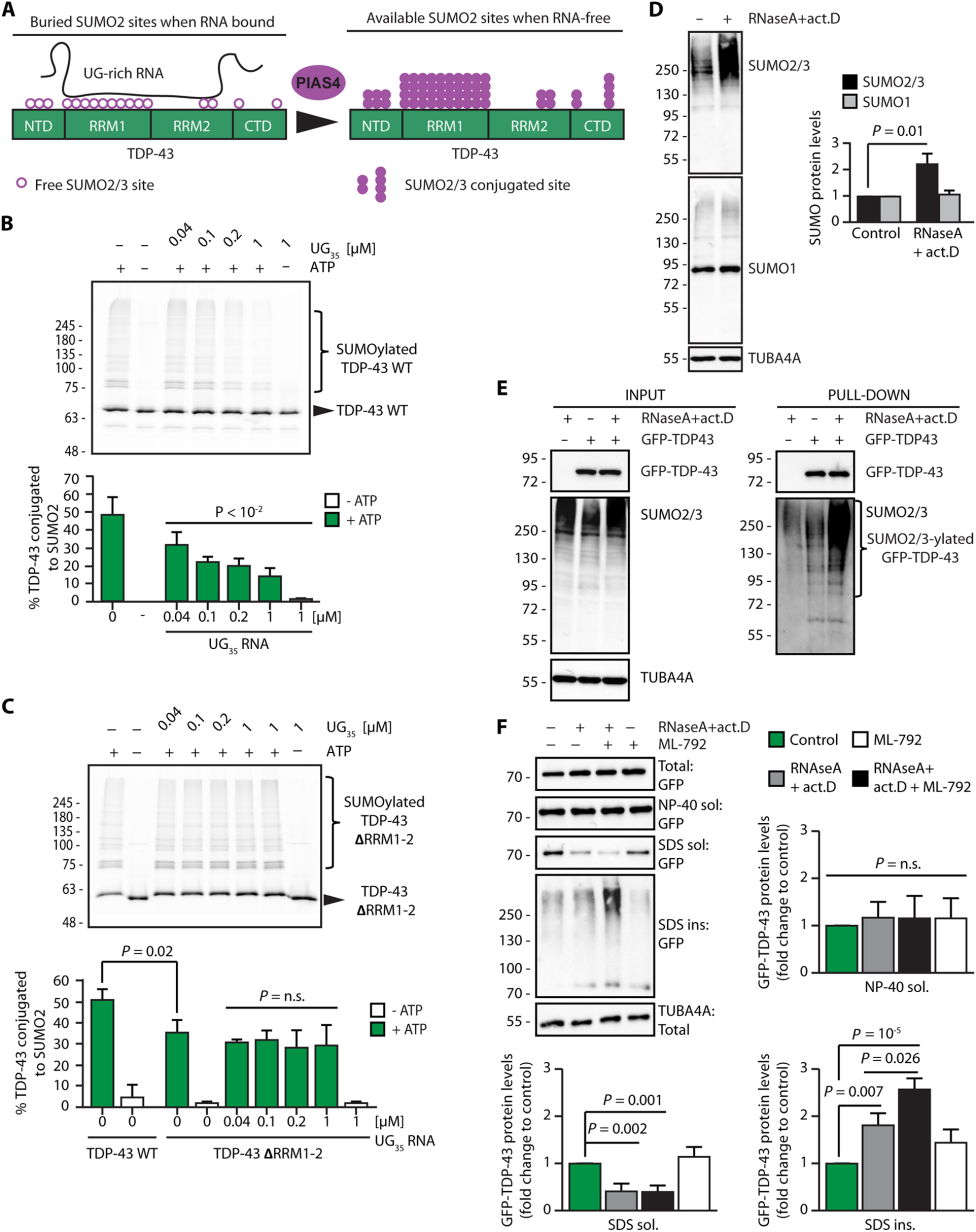
SUMO2/3-ylation protects RNA-unbound TDP-43 from aggregation. (**A**) Schematic model showing that binding of TDP-43 to UG-rich RNA masks the K residues located in the two RRMs, while loss of RNA binding makes them available for SUMO2/3-ylation. (**B**) Representative image and quantification of the percentage of in vitro SUMO2-ylated TDP-43-GFP by PIAS4 in the absence of UG_35_ RNA (column 1) and in the presence of increasing concentrations of UG_35_ RNA (columns 3 to 7). Where indicated, (−) ATP was omitted from the reaction mixture (columns 2 and 7) (mean ± SEM, *n* = 3). One-way ANOVA, followed by Bonferroni-Holm post hoc test. (**C**) Representative image and quantification of the percentage of in vitro SUMO2-ylated TDP-43-GFP-ΔRRM1-2 by PIAS4 in the absence or presence of ATP; where indicated, increasing concentrations of UG_35_ RNA were added; quantification of the SUMO2-ylated TDP-43-GFP-ΔRRM1-2 versus WT is also shown (mean ± SEM, *n* = 3). One-way ANOVA, followed by Bonferroni-Holm post hoc test. (**D**) Immunoblots of U2OS cells untreated or treated with RNase A (10 μM) and actinomycin D (4 μM) for 2 hours (mean ± SEM, *n* = 3). TUBA4A was used as loading control. One-way ANOVA, followed by Bonferroni-Holm post hoc test. (**E**) Immunoprecipitation under denaturing conditions of GFP-TDP-43 from U2OS cells treated as described in (D). Immunoblots of total protein (input) and beads (pull-down) fractions are shown. TUBA4A was used as loading control. (**F**) Sequential fractionation of NP-40 soluble, SDS-soluble, and insoluble proteins from U2OS cells overexpressing GFP-TDP43 and either left untreated or treated with RNase A (10 μM) and actinomycin D (4 μM) for 2 hours, alone or with a pretreatment with ML-792 (2 μM) for 2 hours. Quantification of GFP-TDP-43 levels in the NP-40 soluble, SDS-soluble, and insoluble fractions, expressed as fold change compared to the control condition (mean ± SEM, *n* = 5). TUBA4A was used as loading control. One-way ANOVA, followed by Bonferroni-Holm post hoc test.

We observed that the lysine residues located within the RRM1 domain of TDP-43 are conjugated to SUMO2/3 upon arsenite treatment (Fig. 4). TDP-43 binds to UG-rich RNA via its RRMs (RRM1 and RRM2) (*37*). Binding to UG-rich RNA retains TDP-43 inside the nucleus (*46*) and inhibits TDP-43 aggregation and toxicity (*47*–*49*). RNA binding to proteins involves lysine residues in the RRMs (*36*). We reasoned that bound RNA could mask lysine residues and compete with TDP-43 SUMOylation (Fig. 6A). By contrast, when TDP-43 dissociates from RNA, as during stress conditions that cause TDP-43 accumulation in the cytoplasm, the lysine residues could become available for conjugation with SUMO2/3 (Fig. 6A). Accordingly, stress-induced SUMO2/3-ylation of TDP-43 could represent a backup system that maintains the solubility of TDP-43 molecules that do not tightly bind to RNA. To test this hypothesis, we analyzed PIAS4-mediated TDP-43 SUMOylation in vitro in the absence of UG-rich RNA (Fig. 6B, first column) or in the presence of increasing concentrations of UG-rich RNA (Fig. 6B, columns 2 to 5). SUMO2 conjugation to TDP-43 inversely correlated with the concentration of RNA (Fig. 6B; UG_35_ RNA). As a control, we used a deletion mutant of TDP-43 lacking the two RRMs (ΔRRM1-2). Compared to TDP-43-GFP WT, the degree of PIAS4-mediated SUMO2-ylation of ΔRRM1-2 was lower, supporting the idea that some of the lysine residues located in the RRMs become modified with SUMO2 (Fig. 6C). However, the data also demonstrate that other residues located within the N-terminal and/or C-terminal region of TDP-43 are efficiently modified alongside the residues located within the RRMs, in line with the observed SUMO2/3-ylation of the N 3K/R, RRM1 5K/R, RMM2 2K/R, and C 2K/R variants (Fig. 4, B, E, and F). In vitro PIAS4 efficiently SUMO2-ylated TDP-43-ΔRRM1-2 regardless of increasing concentrations of UG_35_ RNA (Fig. 6C). Together, these data suggest that RNA binding masks the lysine residues located within the RRMs, thus preventing modification of TDP-43 by PIAS4.

### SUMO2/3-ylation is a mechanism that protects RNA-free TDP-43 from aggregation

Recent data using cell lysates show that enzymatic degradation of RNA leads to the aggregation of a large variety of RBPs, including TDP-43 (*47*, *50*). In addition, RNA binding to RBPs can regulate their subcellular localization. In the case of TDP-43, binding to nuclear RNA antagonizes TDP-43 nuclear export and retains it in the nucleus (*46*). Conversely, degradation of RNA and inhibition of transcription with actinomycin D (act.D), which decreases the levels of precursor mRNAs, result in TDP-43 mislocalization to the cytoplasm and favor its aggregation (*51*, *52*). In conjunction with our findings that RNA binding inhibits SUMOylation, we hypothesized that low levels of RNA could induce global protein SUMO2/3-ylation in an attempt to increase the solubility of aggregation-prone RBPs, including TDP-43.

To investigate this possibility, we used different approaches. First, we used a previously characterized TDP-43 variant unable to bind to RNA, referred to as 4FL (F147L/F149L/F229L/F231) (*37*). We overexpressed FLAG-TDP-43 WT or 4FL in His10-SUMO2 U2OS cells, followed by pull-down under denaturing conditions to assess their degree of SUMO2/3-ylation. In contrast to FLAG-TDP-43, the 4FL mutant was extensively conjugated to SUMO2/3 in untreated cells (fig. S6A). Arsenite treatment enhanced the SUMO2/3-ylation of FLAG-TDP-43 to a similar extent of FLAG-TDP-43 4FL (fig. S6B). Together, these data support the idea that RNA binding to RRM1 inhibits PIAS4-mediated SUMO2/3-ylation. Second, we treated cells with RNase A and act.D to deplete RNA. Quantification of the fluorescence intensity of the SYTO RNASelect dye that binds RNA (fig. S6C) and quantitative PCR (qPCR) analysis of the levels of ribosomal RNAs (fig. S6D) confirmed the efficacy of the treatment. The loss of cellular RNA induced a global increase in protein SUMO2/3-ylation (Fig. 6D), including SUMO2/3-ylation of TDP-43 (Fig. 6E). By contrast, we did not observe changes in the expression levels of SUMO1 following treatment of the cells with RNase A and act.D (Fig. 6D). Of note, reduction of total RNA levels did not lead to SG formation, but coincided with mislocalization of TDP-43 to the cytoplasm (fig. S6E, F), where protein aggregation is favored. These data further support the idea that SUMO2/3-ylation prevents TDP-43 aggregation in the cytoplasm.

In light of these data, we next verified whether inhibition of SUMOylation enhances the aggregation of TDP-43 when unbound from RNA. U2OS cells overexpressing GFP-TDP-43 were either left untreated or exposed to RNase A and act.D in the absence or presence of ML-792, followed by sequential fractionation of NP-40 soluble, SDS-soluble, and SDS-insoluble proteins. Treatment with RNase A and act.D decreased the SDS-soluble fraction, while inducing the accumulation of GFP-TDP-43 in the SDS-insoluble fraction (Fig. 6F). This suggests that TDP-43 competes with other substrates for SUMO2/3-ylation upon RNA loss and only a fraction of RNA-free TDP-43 molecules that mislocalizes to the cytoplasm upon RNA removal is efficiently SUMO2/3-ylated and can be protected from aggregation. In agreement with this interpretation, we find that the combined treatment with RNase A, act.D, and ML-792, which inhibits global SUMOylation, further enhanced the accumulation of GFP-TDP-43 in the SDS-insoluble fraction (Fig. 6F). Inhibition of SUMOylation upon RNA depletion also enhanced the aggregation of endogenous TDP-43 (fig. S6G) (*50*). Considering the increased aggregation propensity of the RRM1 K/R variant, which does not bind to RNA and is not efficiently conjugated to SUMO2/3 upon stress, these data support the idea that enhanced SUMO2/3-ylation of TDP-43 represents a backup mechanism that protects a fraction of RNA-free TDP-43 molecules from aggregation.

### Inhibition of SUMO-ylation impairs SG disassembly in iPSC-MNs

**Fig. 7.**
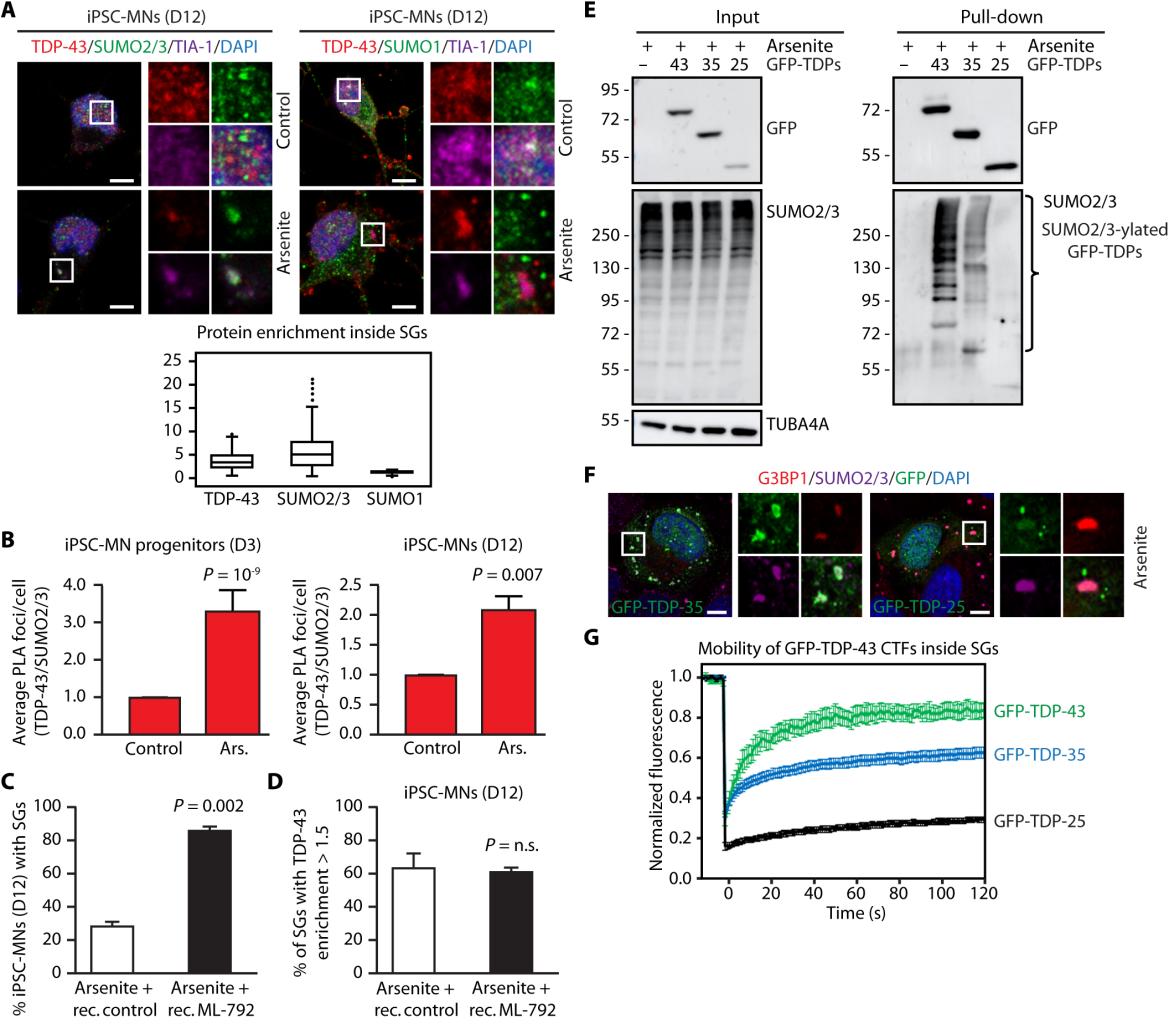
Inhibition of SUMO-ylation affects SG dynamics and TDP-43 mobility in neuronal and ALS patient cells. (**A**) Confocal imaging of endogenous TDP-43, SUMO1, SUMO2/3, TIA-1, and DAPI in iPSC-MNs differentiated for 12 days (D12) and either left untreated or exposed to arsenite (500 μM, 45 min). Average enrichment of TDP-43, SUMO1, and SUMO2/3 inside TIA-1–positive SGs is shown (mean ± SEM, *n* = 215, TDP-43; *n* = 215, SUMO2/3; *n* = 175, SUMO1). Scale bars, 5 μm. (**B**) Percentage of PLA foci/cell in iPSC-MN progenitors (D3) and iPSC-MNs (D12) untreated or arsenite-treated (500 μM, 45 min) and incubated with TDP-43 and SUMO2/3 antibodies (mean ± SEM, *n* = 3). Total number of cells analyzed: *n* = 613 (D3) and 338 (D12), control; *n* = 971 (D3) and 387 (D12), arsenite. One-way ANOVA, followed by Bonferroni-Holm post hoc test. (**C**) Quantification of the percentage of SG-positive iPSC-MNs (D12) that were exposed to arsenite (500 μM, 45 min), followed by recovery in drug-free medium for 2 hours (rec. control) or to a treatment with ML-792 (2 μM) and arsenite, followed by recovery with ML-792 (2 μM) for 2 hours (rec. ML-792). Mean ± SEM, *n* = 3. Student’s *t* test. (**D**) Quantification of the % of persisting SGs with enrichment of TDP-43 higher than 1.5 in iPSC-MNs (D12) treated as described in (C) (mean ± SEM, *n* = 3. Total number of SGs analyzed: 10,859 to 11,896 for SUMO1; 11,345 to 12,296 for SUMO2/3). Fluorescence intensity >1.5 corresponds to SGs with moderate to high enrichment of TDP-43. Student’s *t* test. (**E**) Immunoprecipitation under denaturing conditions of overexpressed GFP-tagged TDP-43, TDP-35, and TDP-25 in U2OS cells treated with arsenite. Immunoblots of total protein (input) and beads (pull-down) fractions are shown. TUBA4A was used as loading control. (**F**) Confocal imaging of GFP-tagged TDP-35 and TDP-25, G3BP1, SUMO2/3, and DAPI in arsenite-treated U2OS cells. Scale bars, 5 μm. (**G**) FRAP curves of overexpressed GFP-tagged TDP-43, TDP-35, and TDP-25 in U2OS cells treated with arsenite (mean ± SEM, *n* = 6, GFP-TDP-43; *n* = 15, GFP-TDP-35; *n* = 9, GFP-TDP-25).

TDP-43 inclusions in ALS and ALS-FTD mainly accumulate in neurons in the brain and spinal cord, although they can also be found in glial cells (*53*, *54*), intramuscular nerve bundles (*55*), and skeletal and cardiac muscle cells (*56*). We thus tested the impact of SUMOylation inhibition on TDP-43 mobility in disease-relevant cells. SUMO2/3 was conjugated to GFP-TDP-43 also in neuronal-like SH-SY5Y cells exposed to arsenite (fig. S7A). Cell pretreatment with ML-792 inhibited the SUMO2/3-ylation of GFP-TDP-43 (fig. S7A) and led to its immobilization inside SGs (fig. S7B). We then used induced pluripotent stem cells (iPSCs) that express the inducible transcription factors Ngn2, Isl1, and Lhx3 (NIL) and efficiently generate spinal MNs (iPSC-MNs) (*57*), as shown by the down-regulation of the pluripotency markers OCT4 and NANOG and the progressive induction of the neuronal markers TUJ1 and ISL1 in iPSC-MN progenitors and iPSC-MNs differentiated for 3 and 12 days, respectively (fig. S7, C and D). Endogenous SUMO2/3 and TDP-43 colocalized inside SGs in arsenite-treated iPSC-MNs, regardless of the differentiation stage (fig. S7E, progenitors; Fig. 7A, mature). By contrast, SUMO1 was not enriched inside SGs in arsenite-treated iPSC-MNs (Fig. 7A). We also confirmed colocalization of PIAS4 with G3BP1 inside arsenite-induced SGs in iPSC-MN progenitors (fig. S7F). Of note, in iPSC-MN progenitors treated with arsenite, we also observed a decrease of the nucleus/cytoplasm ratio of PIAS4, compared to the untreated cells (fig. S7G), and an enrichment of PIAS4 in more than 60% of the induced SGs (fig. S7H). We then investigated by PLA whether arsenite treatment increases the proximity between endogenous TDP-43 and SUMO2/3 in iPSC-MNs differentiated for 3 and 12 days, respectively. Compared to the untreated iPSC-MNs, the number of PLA foci/cell significantly increased following arsenite treatment, indicating enhanced interaction/conjugation of SUMO2/3 to TDP-43 (Fig. 7B), similar to what was observed in U2OS cells (fig. S2G). Moreover, inhibition of SUMO-ylation significantly delayed the disassembly of arsenite-induced SGs during the stress recovery phase in both iPSC-MN progenitors (fig. S7I) and iPSC-MNs (Fig. 7C). We noticed that during the stress recovery phase, the persisting SGs contained a similar amount of TDP-43 regardless of the pretreatment with ML-792 (Fig. 7D). However, SUMOylation inhibition enhanced the number of iPSC-MNs with persisting SGs that contain TDP-43 (Fig. 7C). Altogether, these data show that recruitment of SUMO2/3 inside SGs and accumulation of TDP-43–positive SGs upon inhibition of SUMOylation are independent on the cell type (fig. S2B) and occur regardless of the differentiation stage of iPSC-MNs.

### Decreased SUMO2/3-ylation of the TDP-43 aggregation-prone C-terminal fragments

Besides full-length ubiquitinated and phosphorylated TDP-43, the inclusions found in patients with ALS and ALS-FTD also contain highly aggregation-prone and toxic C-terminal fragments (CTFs), referred to as TDP-35 and TDP-25 (*53*, *58*, *59*). We verified the extent of SUMO2/3-ylation of the TDP-43 CTFs. Of note, while TDP-35 retains both RRM1 and RRM2, TDP-25 only retains a portion of RRM2 and lacks the majority of the SUMO2 identified sites (fig. S7J) (*12*, *13*). Pull-down under denaturing conditions showed reduction and almost complete loss of SUMO2/3 conjugation for TDP-35 and TDP-25 (Fig. 7E). In line with previous findings (*60*), both CTFs were recruited into arsenite-induced SGs, although TDP-25 mainly formed cytoplasmic aggregates devoid of SUMO2/3 (Fig. 7F). We then measured by FRAP the mobility of the CTFs inside SGs. We found that the lower the SUMO2/3-ylation, the lower the protein mobility inside SGs (Fig. 7G). These data support the correlation between TDP-43 SUMO2/3-ylation and aggregation propensity upon stress.

### The presence of TDP-43 cytoplasmic inclusions correlates with reduced cytoplasmic PIAS4 levels in ALS spinal cord α-motor neurons

**Fig. 8.**
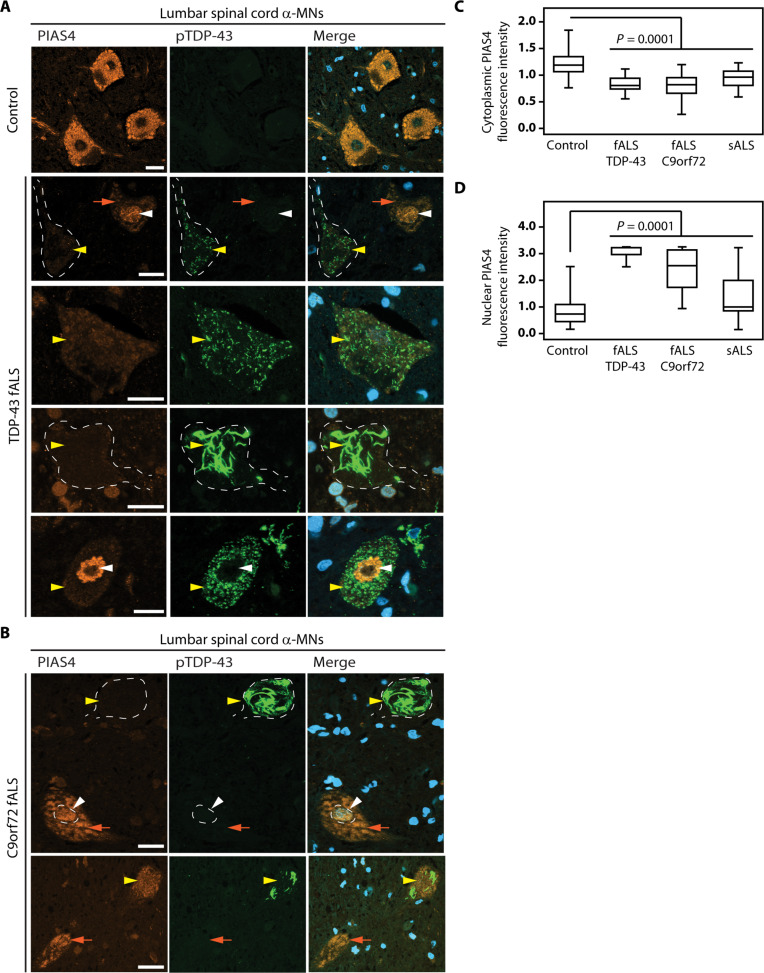
Reduced cytoplasmic PIAS4 immunoreactivity in human ALS lumbar spinal cord α-MNs with TDP-43 pathology. (**A** and **B**) Double immunofluorescence labeling performed on control and fALS (with mutations in the gene coding for TDP-43 and C9orf72, respectively) lumbar spinal cord α-MNs using PIAS4 and phospho Ser409/410 TDP-43 (pTDP-43) antibodies showing a variable pattern of immunoreactivity. Note the overall reduced cytoplasmic PIAS4 immunoreactivity and increased nuclear PIAS4 immunoreactivity (white arrowheads) in fALS α-MNs. α-MNs harboring pTDP-43 aggregates showed markedly reduced levels of PIAS4 (yellow arrowheads). fALS α-MNs showing moderately elevated levels of PIAS4 were devoid of pTDP-43 aggregates (orange arrows). Scale bars, 30 μm (**C** and **D**) Quantification of PIAS4 immunoreactivity in lumbar spinal cord MNs [as shown in (A) and (B)]. Number of α-MNs counted: 992 from four age-matched controls; 159 from two patients with fALS TDP-43; 427 from five patients with fALS C9orf72; 528 from eight patients with sALS. One-way ANOVA, followed by Brown-Forsythe and Welch ANOVA tests, multiple comparisons, *P* = 0.0001 compared to control cases, using GraphPad Prism6 software.

We next sought to investigate whether PIAS4 expression levels and subcellular distribution are altered in patients with fALS who show TDP-43 pathology. We compared familial TDP-43 mutation cases with familial C9orf72 cases and normal age-matched controls as a reference (table S1). fALS cases were chosen to specifically minimize the variability among sporadic ALS cases. In control cases, the lumbar spinal cord α-motor neurons (α-MNs) exhibited overall substantial cytoplasmic PIAS4 immunoreactivity and low nuclear immunoreactivity (Fig. 8A). By contrast, lumbar spinal cord α-MNs from TDP-43 and C9orf72 fALS cases showed a significant reduction in cytoplasmic PIAS4 immunoreactivity (Fig. 8, A to D), accompanied by a strong increase in nuclear immunoreactivity (Fig. 8, A to D, white arrowheads). Notably, in both TDP-43 and C9orf72 fALS cases, α-MNs characterized by the presence of cytoplasmic aggregates positive for phosphorylated TDP-43 had reduced cytoplasmic PIAS4 immunoreactivity (Fig. 8, A and B, yellow arrowheads), whereas α-MNs with increased cytoplasmic PIAS4 immunoreactivity often lacked TDP-43 aggregates (Fig. 8, A and B, orange arrows). This inverse correlation between cytoplasmic PIAS4 immunoreactivity and TDP-43 aggregation was also consistently observed in sporadic ALS cases (fig. S8). These data suggest that, in the fALS and sALS cases investigated, the reduced cytoplasmic levels of PIAS4 could contribute to enhance TDP-43 aggregation by decreasing its SUMO2/3-ylation.

## DISCUSSION

TDP-43 dysfunction has been linked to a growing number of neurodegenerative diseases, including ALS, FTD, Alzheimer’s disease (*61*), and in the aging brain, where TDP-43 pathology is referred to as limbic-predominant, age-related TDP-43 encephalopathy (*54*). In this study, we show that upon oxidative stress, PIAS4 modifies TDP-43 with SUMO2/3 to increase the solubility of TDP-43 and prevent its aggregation in the cytoplasm. Binding of TDP-43 to UG-rich RNA antagonizes PIAS4-mediated SUMO2/3-ylation, while RNA dissociation promotes TDP-43 SUMO2/3-ylation. Combined loss of RNA binding and defective SUMO2/3-ylation promote TDP-43 aggregation. Collectively, our data suggest that conjugation of TDP-43 with SUMO2/3 is a rescue mechanism to control the aggregation of RNA-free TDP-43 in the cytoplasm and prevent the formation of pathological TDP-43 aggregates.

Previous studies showed that TDP-43 aggregates are modified with several PTMs, including ubiquitination, phosphorylation, and proteolytic processing (*53*, *62*). Ubiquitinated fragments of TDP-43 can exert toxicity, if not removed by the degradation machineries. Initial studies suggested that phosphorylation of TDP-43 is linked to its protein aggregation (*62*, *63*). However, recent evidence shows that phosphorylation enhances TDP-43 solubility inside condensates (*6*). This suggests that PTMs on TDP-43 may be an attempt by the cell to maintain the solubility of TDP-43 under unfavorable conditions, such as, for example, oxidative stress.

Previous work showed that TDP-43 inclusions can also contain SUMO2/3 (*64*); moreover, TDP-43 can be conjugated to SUMO1 at the lysine residue 136, which promoted the nuclear retention of TDP-43, indirectly reducing its cytoplasmic aggregation (*9*, *10*). TDP-43 can also be modified with mixed SUMO2/3-polyubiquitin chains in mammalian cells exposed to heat shock (*18*). Yet, how exactly SUMO2/3 influences TDP-43 stability and function is largely unknown.

**Fig. 9.**
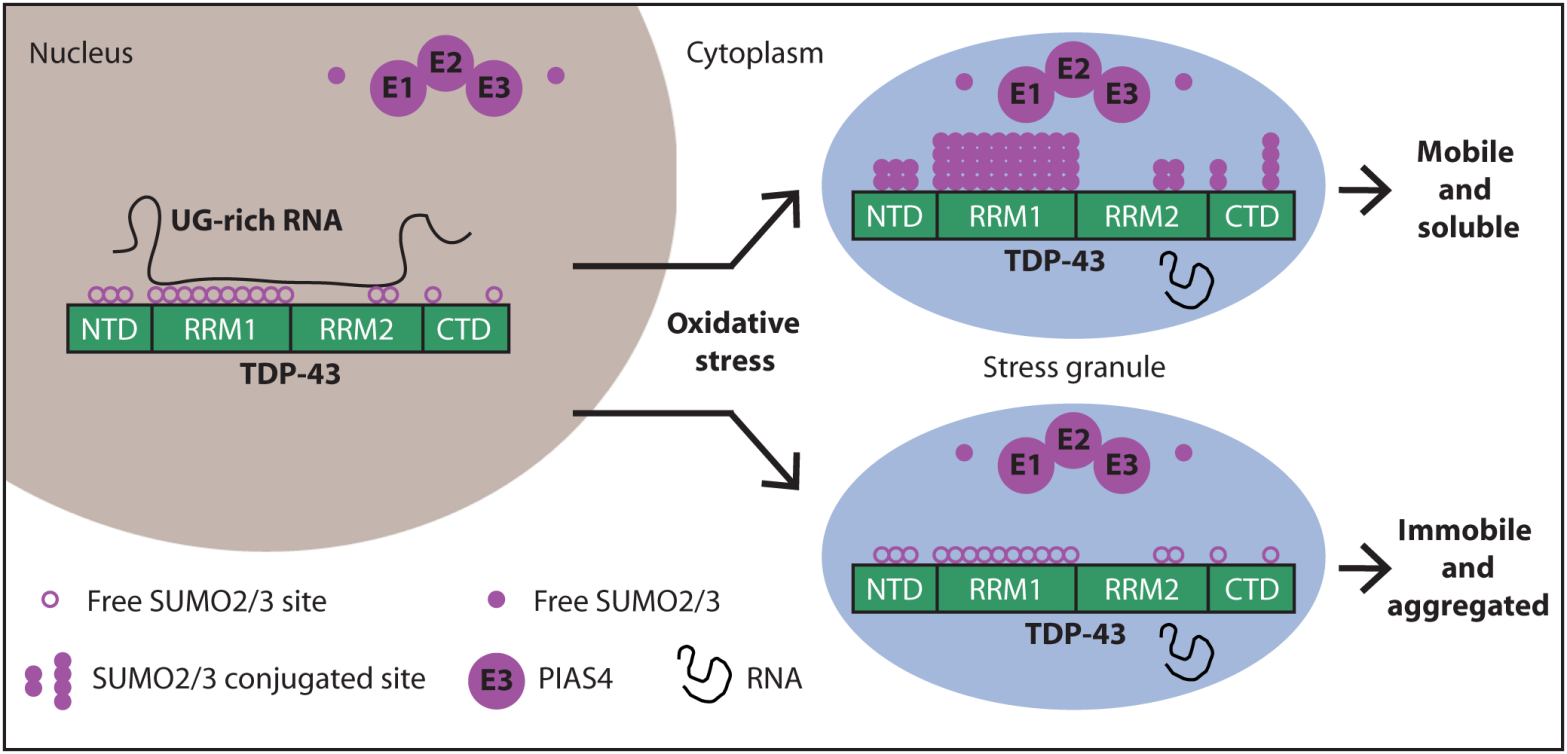
SUMO2/3-ylation protects RNA-free TDP-43 from aggregation upon oxidative stress. When cells are not subjected to stress, TDP-43 is mainly localized inside the nucleus, where it binds with high affinity to UG-rich RNA. RNA masks the K residues located in the RRM1 and RRM2, reducing the access to PIAS4. Upon oxidative stress, a fraction of TDP-43 translocates into the cytoplasm, where it localizes inside SGs along with the SUMO machinery (free SUMO2/3, E1, E2 and E3 enzymes, including PIAS4). RNA-free TDP-43 molecules can be efficiently conjugated to SUMO2/3 by PIAS4, thereby protecting TDP-43 from immobilization inside SGs and aggregation.

Our study provides evidence of TDP-43 SUMO2/3-ylation as a mechanism to decrease the aggregation of TDP-43 upon oxidative stress (Fig. 9). Although TDP-43 conjugation to SUMO2/3 can occur both in the nucleus and in the cytoplasm, our data suggest that the cytoplasmic pool of RNA-free TDP-43 is preferentially conjugated with SUMO2/3 chains (Figs. 1 and 2). The combined data suggest that SUMO2/3-ylation serves as a backup mechanism to maintain TDP-43 solubility under unfavorable conditions such as low levels of RNAs, as they occur in the cytoplasm (*47*). In agreement, the RRM1 10K/R SUMO2/3-ylation defective mutant that is also unable to bind to RNA shows enhanced aggregation propensity (Fig. 4 and fig. S4).

Previous work showed that in response to stress, TDP-43 is recruited into nuclear condensates that lack RNA and have been termed anisosomes (*65*). TDP-43 recruitment into nuclear anisosomes is promoted by acetylation of TDP-43 on K145, K136, and K192, which impairs RNA binding to TDP-43 (*66*–*68*). Anisosomes are uneven bodies with an inner core enriched for the heat shock protein 70 (HSP70) chaperones and an outer core containing TDP-43. The ATP-dependent chaperone activity of HSP70s is required to maintain TDP-43 solubility inside anisosomes during stress (*65*). Where exactly TDP-43 acetylation takes place is unknown; RNA-free acetylated TDP-43 constantly moves from the nucleoplasm into the anisosomes, where HSP70s prevent TDP-43 aggregation (*65*). Thus, the nucleus harbors a specific type of condensate that appears to function as a compartment to keep RNA-free acetylated TDP-43 soluble. By contrast, in the cytoplasm, RNA-free acetylated TDP-43 is more vulnerable to aggregation (*66*). Our data suggest that SGs serve a similar function to anisosomes in controlling the solubility of TDP-43 and presumably other RBPs in the cytoplasm. Conjugation of TDP-43 with SUMO2/3 maintains its mobility inside SGs, and the concentration of SUMO2/3 is high inside SGs, compared to the surrounding cytoplasm. Moreover, the Ubc9 E2 conjugating enzyme, along with the E3 ligases PIAS4 (this study) and RanBP2 (*19*), are recruited inside SGs. This suggests that SGs could be dedicated cytosolic compartments for the quality control of aggregation-prone RBPs. Because acetylation and SUMOylation can compete for the same residues, it is tempting to speculate that SGs may indirectly help shift the balance from RNA-free TDP-43 acetylation to SUMO2/3-ylation, preventing irreversible aggregation. Future studies should address the question of how cells use acetylation, SUMO1, or SUMO2/3-ylation to control TDP-43 stability in the nucleus and cytoplasm and how imbalances in the competitive mechanisms between these PTMs contribute to TDP-43 aggregation.

Although SUMO2 sites have been identified in the N and C termini of TDP-43, the majority is located within the RRM1 (*13*) and is hidden when TDP-43 is bound to RNA (Fig. 6A). In agreement, RNA-free TDP-43 exposes lysine residues that have been identified previously as SUMO2 sites (*13*) and RNA decrease enhances TDP-43 SUMO2/3-ylation (Fig. 6). This idea is further substantiated by the finding that the RNA-binding deficient 4FL variant is constitutively conjugated to SUMO2/3 in untreated cells (fig. S6A). These data suggest that the lysine residues located within the RRM1 are targeted by SUMO2/3-ylation to prevent its irreversible aggregation inside SGs. This idea is further substantiated by the finding that the K/R substitutions within the RRM1 domain (RRM1 10K/R), which abrogate RNA binding and conjugation to SUMO2/3, enhanced TDP-43 aggregation in the cytoplasm and within SGs.

The role of SGs in the process of TDP-43 aggregation has been debated. Some studies have proposed that TDP-43 aggregation can occur independently of SGs (*15*, *69*), others have reported that SGs serve as a seed for protein aggregation (*70*, *71*), and others have proposed that SGs are protective sites (*48*, *72*). Our data are consistent with the latter proposal. Considering that TDP-43 shuttles from the cytoplasm into SGs, we cannot exclude the possibility that SUMO2/3-ylation may occur at the border of SGs. However, given that SUMO2/3-ylation maintains TDP-43 solubility and mobility inside SGs, we propose that SGs act as a compartment that can protect RNA-free TDP-43 from aggregation by promoting and/or enriching its SUMO2/3-ylation (Fig. 9). In agreement with this idea, the SUMO2/3 and SG proteomes largely overlap (*19*) and previous work has identified SUMO2 sites in several RBPs that are recruited inside SGs, such as G3BP1, hnRNPs, and FUS (*13*). To what extent these RBPs are conjugated to SUMO2/3 during stress and whether their SUMO2/3-ylation is a strategy to maintain their solubility warrants future investigations.

The ability of SGs to buffer TDP-43 aggregation likely depends on several factors. First, SGs contain large amounts of RNA, along with many different RBPs that can compete for binding to RNA. Although TDP-43 has been shown to bind with less affinity to various RNA binding sequences (*73*), it is rather selective for RNAs with long UG-rich sequences, which are enriched in human cryptic exons (*74*); binding to its specific RNA targets, rather to any type of RNA, stabilizes TDP-43 (*37*, *75*). The low abundance of UG-rich RNA in the cytoplasm, compared to the nucleus, could promote TDP-43 aggregation, regardless of the global RNA concentration within SGs, but this remains to be tested. Second, the efficiency of SUMO2/3-ylation can be limited by the overall low stoichiometry of SUMO (*76*) and the local concentration of TDP-43 and the E3 ligase PIAS4. Conditions that decrease the efficiency of TDP-43 SUMO2/3-ylation or lead to the accumulation inside SGs of high amounts of substrates that compete with each other’s for SUMO2/3-ylation would overwhelm this protective mechanism. For example, defective ribosomal products (DRiPs) are released in large amounts upon polyribosome disassembly, and their accumulation inside SGs promotes their conversion into a less dynamic, aggregated state (*77*, *78*). The molecular chaperones VCP and the HSPB8-BAG3-HSP70 complex prevent this from happening, whereas dysfunction of these chaperones promotes SG aggregation (*77*–*79*). Upon stress, DRiPs are rapidly SUMO2/3-ylated by the cells (*80*). Thus, conditions that impair the protein quality control system could promote the conversion of SGs from a cytoprotective site into a pro-aggregation site at least in part by inducing the accumulation inside SGs of DRiPs, which can compete with TDP-43 for efficient SUMO2/3-ylation.

Although our data demonstrate that SGs concentrate the SUMO machinery during stress, SUMO2/3-yation was also observed upon inhibition of RNA synthesis, which, per se, does not elicit the formation of SGs, indicating that SGs do not appear to be essential for TDP-43 SUMO2/3-ylation (Fig. 6 and fig. S6). Together, these data demonstrate that TDP-43 SUMO2/3-ylation is a mechanism activated by the cells, including MNs at various stages of differentiation, to protect RNA-free TDP-43 from irreversible aggregation. Understanding these molecular mechanisms may have important therapeutic applications. We find that the highly aggregation-prone pathological TDP-43 fragments are poorly conjugated to SUMO2/3. Moreover, the subcellular distribution of PIAS4 is altered in the human spinal cord of fALS cases, where its cytoplasmic levels decrease and correlate with the presence of cytoplasmic TDP-43 inclusions. Because PIAS4 is the preferred E3 ligase for TDP-43 and its activity is outcompeted by the presence of RNA, our data suggest that a decrease in PIAS4 levels in the cytoplasm of MNs may increase TDP-43 vulnerability to aggregation. Future studies are required to determine whether (i) the altered subcellular distribution of PIAS4 may affect MN’s ability to efficiently conjugate SUMO2/3 to TDP-43, thereby influencing TDP-43 aggregation in the cytoplasm; (ii) SUMOylation activators that show promising neuroprotective properties (*81*–*84*) may enhance PIAS4-dependent SUMO2/3-ylation of TDP-43, thereby decreasing its aggregation.

## MATERIALS AND METHODS

### Cell lines, culture conditions, and differentiation to motor neurons

HeLa Kyoto cells (*85*), U2OS cells, His10-SUMO2 U2OS cells, human embryonic kidney (HEK) 293T cells stably expressing His6-SUMO2, and SH-SY5Y cells were grown at 37°C and 5% CO_2_ in Dulbecco’s modified Eagle’s medium (DMEM) with high glucose (4.5 g/liter) supplemented with 2 mM l-glutamine, penicillin/streptomycin (100 U/ml), and 10% fetal bovine serum. U2OS G3BP1/2 KO cells were a gift from N. Kedersha. iPSCs (WTSIi004-A, EBiSC—European Bank for induced pluripotent Stem Cells) expressing inducible NIL for differentiation into spinal motor neurons were described elsewhere (*57*). iPSCs were grown on cell culture plates coated with Matrigel LDEV-Free hESC-Qualified (Corning), using NutriStem hPSC XF medium with 0.1× penicillin-streptomycin. Cells were passaged every 3 to 5 days with Dispase (1 mg/ml). For differentiation to MN progenitors (day 3) and mature MNs (day 12), we followed previously published protocols (*57*, *86*).

### Caenorhabditis elegans

Hermaphrodite worms were maintained at 20°C on nematode growth medium (NGM) agar plates seeded with *Escherichia coli* strain OP50. NGM plates contain 1.5% agar, 0.25% tryptone, 0.3% sodium chloride, 1 mM calcium chloride, 1 mM magnesium sulfate, 25 mM potassium phosphate (pH 6.0), and cholesterol (5 μg/ml). The DE115 (dnSi8 [tdp1::flag::mCherry + Cbr-unc119(+)] CGC) strain expressing tdp1-mCherry fusion and sup-46-GFP strain (*35*) was used in this study.

### Plasmids, siRNA, transfection, and cell treatments

Cells were lipofected with siRNAs and plasmids using Lipofectamine 2000 and 3000 (Life Technologies), respectively, following manufacturer instructions. Twenty-four hours after transfection of plasmids or 72 hours after transfection of siRNAs, cells were processed for immunoblotting, RT-qPCR, or live imaging. *PIAS4* was synthesized via GenScript, then cloned into the vector pOCC151-pOEM1-GST-PS-ccdB for expression and purification of GST-tagged PIAS4. Constructs were sequence verified using Sanger sequencing.

The DNAs coding for the GFP-TDP-43 K/R variants and the flag-siRNA–resistant TDP-43 K/R variants described in this study were generated via GENEWIZ (from Azenta Life Sciences) using GFP-TDP-43 WT and pCMV4flagsiRTDP-43 WT as templates, respectively.

To induce SGs, cells were treated with sodium arsenite (500 μM for 45 min to 1 hour) the day after seeding (for nontransfected cells), or 24 and 72 hours after transfection of plasmids and siRNA, respectively. To inhibit SUMOylation, ubiquitination, and NEDDylation, the following inhibitors were used: ML-792, TAK-243 (MLN7243), and MLN4924. RNA depletion was achieved by treating the cells with RNase A and act.D. The concentration and treatment duration are reported in the figure legends.

### Immunoprecipitation assay and isolation of His10-SUMO2 conjugates by Ni-NTA pull-downs under denaturing conditions

For immunoprecipitation of GFP-tagged proteins under denaturing conditions, cells were treated as indicated in the figure legends. Cells were lysed in lysis buffer [150 mM tris-HCl, pH 6.7, 5% SDS, 30% glycerol, 20 mM *N*-ethylmaleimide (NEM), and complete EDTA]; cell lysates were then diluted 1:10 in phosphate-buffered saline (PBS) 1× supplemented with 0.5% NP-40. Lysates were sonicated and centrifuged at 12,000*g* for 10 min at 4°C, followed by incubation with equilibrated GFP-Trap beads for 1 hour at 4°C. After incubation, the immune complexes were centrifuged at 2500*g* for 5 min at 4°C. Beads were washed three times with wash buffer (10 mM tris-Cl, pH 7.5, 150 mM NaCl, 0.05% NP-40, and 0.5 mM EDTA). Both immunoprecipitated proteins and input fractions were resolved on SDS–polyacrylamide gel electrophoresis (SDS-PAGE) and analyzed by Western blotting.

Isolation of His-tagged SUMO2-conjugated proteins under denaturing conditions was performed using His10-SUMO2 U2OS cells. Briefly, cells were lysed in 6 M guanidinium-HCl, 0.1 M Na_2_HPO_4_/NaH_2_PO_4_, 0.01 M tris-HCl, pH 8.0, 5 mM imidazole, and 10 mM β-mercaptoethanol. Ni-NTA-agarose beads (Qiagen) were then added and lysates were rotated at room temperature (RT) for 2 hours. The beads were then washed for 5 min, using the following buffers: 6 M guanidinium-HCl, 0.1 M Na_2_HPO_4_/NaH_2_PO_4_, 0.01 M tris-HCl, pH 8.0, plus 10 mM β-mercaptoethanol and 0.1% Triton X-100; 8 M urea, 0.1 M Na_2_HPO_4_/NaH_2_PO_4_, 0.01 M tris-HCl, pH 8.0, 10 mM β-mercaptoethanol, and 0.1% Triton X-100 (1×); 8 M urea, 0.1 M Na_2_HPO_4_/NaH_2_PO_4_, 0.01 M tris-HCl, pH 6.3, 10 mM β-mercaptoethanol, and 0.1% Triton X-100 (3×). After the last wash, His10-tagged SUMO2-conjugated proteins were eluted by incubating the beads in 200 mM imidazole, 0.15 M tris-HCl, pH 6.7, 30% glycerol, 0.72 M β-mercaptoethanol, and 5% SDS for 20 min at RT.

### Coimmunoprecipitation

For immunoprecipitation under nondenaturing conditions, cells were harvested in lysis buffer (50 mM tris-HCl, pH 7.4, 150 mM NaCl, 1 mM EDTA, 1% Triton X-100, 20 mM NEM, and RNase) and lysed by passing through a 26G needle three times. The cell lysates were incubated on ice for 20 min, then centrifuged at 10,000*g* at 4°C for 10 min. A fraction of the supernatant was kept as input fraction, while the rest was incubated with preequilibrated FLAG M2 magnetic beads (Sigma Aldrich) for 1 hour at 4°C. The supernatant was removed with a magnetic separator and the beads were washed three times with TBS (50 mM tris-HCl, pH 7.4, and 150 mM NaCl). Coimmunoprecipitated proteins were recovered using Laemmli buffer. All protein fractions were boiled at 95°C for 3 min to elute the bound proteins, resolved on SDS-PAGE, and analyzed by Western blotting.

### Isolation of SGs

SGs were prepared according to Wheeler *et al.* (*27*). Briefly, 80% confluent HEK293 cells (~25 × 10^6^) expressing His6-SUMO2 were either untreated or treated with sodium arsenite (0.5 mM) for 1 hour. Cells were then washed with fresh media, scraped in media, and collected in 5 ml. An aliquot of 100 μl was kept as input and the remaining volume was centrifuged at 1700*g* for 5 min. The cell pellet was flash frozen in liquid nitrogen. The pellet was then thawed on ice in 900 μl of lysis buffer [50 mM tris-HCl, pH 7.4, 100 mM potassium acetate, 2 mM magnesium acetate, 0.5 mM DTT, heparin (50 μg/ml), 0.5% NP-40, and 1:5000 Antifoam emulsion, supplemented with complete no EDTA tablet inhibitors (Roche) and RNAsin 0.25 U/μl] and cells were disrupted by passage through a 25G needle and two cycles of sonication (10 s on/30 s off). Debris was removed by centrifugation for 5 min at 900*g*. Supernatant was then centrifuged at 20,000*g* for 20 min and the SG containing pellet was washed by resuspension in 400 μl of lysis buffer and spun again 20,000*g* for 20 min. The obtained pellet was then resuspended in 100 μl of lysis buffer and centrifuged for 2 min at 900*g* at 4°C to remove any remaining debris. The supernatant containing SGs was centrifuged at 20,000*g* for 20 min at 4°C. The pellet containing SG was resuspended in 500 μl of 8 M urea, pH 8, 5 mM imidazole, and 10 mM β-mercaptoethanol, and 100 μl was kept for input of the SG isolated fraction. The pellet was resuspended in 8 M urea, followed by Western blotting.

### Protein solubility assay and Western blotting

For protein solubility assays, cells were lysed in a buffer containing 0.5% NP-40 (20 mM tris-HCl, pH 7.4, 150 mM NaCl, 1.5 mM MgCl_2_, 3% glycerol, and 0.5% NP-40) supplemented with protease inhibitors and 1 mM DTT. Cells were lysed by passing through a 26G needle three times, kept on ice for 30 min, and then centrifuged at 10,000*g* at 4°C for 15 min. The supernatant was collected as NP-40 soluble fraction, while the pellet was resuspended with 2% SDS Laemmli buffer, sonicated, and centrifuged at 10,000*g* at 4°C for 20 min. The supernatant was collected as SDS-soluble fraction; the pellet was incubated with formic acid at 37°C for 35 min and left overnight in a SpeedVac vacuum concentrator at 45°C, followed by resolubilization with 2% SDS Laemmli buffer. Samples were sonicated. The solubility assay of endogenous TDP-43 upon RNA depletion was performed as previously reported (*50*). Briefly, cells were either left untreated or exposed to treatment with RNase A and act.D for 2 hours; where indicated, cells were pretreated with ML-792 for 2 hours. Then, cells were lysed with lysis buffer (20 mM tris-HCl, pH 7.5, 150 mM NaCl, 3 mM EDTA, 1% Triton, 0.5% Na-deoxycholate, complete EDTA, and 1 mM DTT) supplemented with 20 mM NEM, by passing through a 26G needle three times, followed by sonication. A fraction of the total lysate was kept. The lysate was then centrifuged at 21,000*g* at 4°C for 30 min and the supernatant was incubated at 37°C for 30 min in the absence or presence of RNase A, act.D, and ML-792 as previously reported (*50*). The supernatant was then centrifuged at maximum speed for 30 min to fractionate the soluble proteins from the pellet. The centrifuged supernatant was kept as the soluble protein fraction. The pellet was resolubilized with a buffer containing 20 mM tris-HCl, pH 7.5, 2% SDS, 8 M urea, and 20 mM NEM and sonicated before analysis via Western blotting, as previously published (*50*). For immunoblotting of total protein extracts, cells were harvested in Laemmli sample buffer (supplemented with 20 mM NEM), followed by sonication. Total protein samples and protein fractions were supplemented with β-mercaptoethanol, boiled for 3 min at 100°C, followed by SDS-PAGE and transfer onto nitrocellulose membranes. Protein levels were analyzed by Western blotting using specific antibodies.

### Immunofluorescence microscopy and PLA on cells

Cells were grown on poly-l-lysine–coated glass coverslips and treated as indicated in the figure legends. SGs were induced by treating the cells with 500 μM sodium arsenite for 45 min. Inhibition of SUMOylation was achieved by treating the cells for 2 hours with 2 μM ML-792 alone or followed by incubation with sodium arsenite for additional 45 min. Total RNA levels were decreased by cotreating the cells with 5 μM act.D and 10 μM RNase A for 2 hours. Untreated and treated cells were washed with cold PBS, followed by fixation with ice-cold methanol for 5 min at −20°C. Alternatively, cells were fixed with 3.7% formaldehyde in PBS for 9 min at RT, followed by permeabilization with ice-cold acetone for 5 min at −20°C. Cells were then incubated with a blocking solution (PBS supplemented with 3% BSA and 0.1% Triton X-100) for 1 hour at RT, and subsequently incubated with blocking solution containing primary antibodies at 4°C overnight. Cells were washed three times with PBS, incubated with secondary antibodies and DAPI at RT before mounting. PLA was performed with the Duolink In Situ Red Kit (Sigma-Aldrich), using the indicated antibodies and following manufacturer instructions.

### High-content imaging-based assay

Images were obtained with a Leica SP8 confocal microscope using a 63× oil immersion objective. SUMO2/3 or PIAS4 enrichment inside SGs were analyzed using the ScanR Analysis software (Olympus). First, SGs were segmented based on G3BP1 signal using edge detection algorithm. Following the segmentation, we measured the mean fluorescence intensity of the protein of interest in each detected G3BP1-positive SG. In addition, the mean fluorescence intensity of the protein was measured in an area surrounding it. The relative enrichment of the protein in individual SGs was calculated as a ratio of the mean fluorescence intensity inside divided by the mean intensity in the region surrounding the structure. The values were plotted as column graphs representing the fractions divided by their range values.

Colocalization between TDP-43 and SUMO1 or SUMO2/3 inside nuclei or SGs was performed using Fiji’s plugin Coloc2. Pearson’s coefficient was calculated between TDP-43 and SUMO channels applying a mask for nuclei based on DAPI channel or for SGs based on TIA-1 channel.

Endogenous TDP-43 subcellular distribution in the nucleus and cytoplasm was calculated using the ScanR software (Olympus). Briefly, nuclei were segmented based on DAPI signal using an intensity detection algorithm. Then, TDP-43 mean fluorescence intensity was calculated inside the nucleus and in the region surrounding the nucleus, by applying a fixed distance in pixels from the segmented nucleus.

Quantification of the fluorescence intensity of SYTO RNASelect in the nucleus was calculated using the ScanR software (Olympus). Nuclei were segmented based on DAPI signal using an intensity detection algorithm.

### *C. elegans* RNAi

RNAi was performed following the feeding method with some modifications (*87*, *88*). For *ubc-9* RNAi, 300 bp of the N-terminal *ubc-9* gene was cloned into the L4440 vector with the following primers: 5′ to 3′ forward: TATATAGCGGCCGCATGTCGGGAATT-GCTGCAGG; 5′ to 3′ reverse: ATATATGCGGCCGCTTGAATTCCAATGAGAAGTTGC. L4 larvae stage worms were individually picked on NGM plates seeded with RNAi bacteria and incubated for 24 hours and the resulting young adults were analyzed.

### Worm live imaging preparation and FRAP

For the arsenite treatment, unseeded NGM plates were soaked with sodium arsenite to achieve a final concentration of 5 or 10 mM. Arsenite-coated plates were then seeded with bacteria, and young adults were placed on these plates for 5 hours. For FRAP acquisitions, young adult (L4) worms were picked individually and placed on an unseeded NGM plate to wash off bacteria and mounted on 2% (w/v) agarose pads and immobilized using 25 mM levamisole in M9 buffer. Acquisitions were performed immediately after (less than 5 min). Imaging and FRAP measurements in *C. elegans* were performed with a confocal microscope Zeiss LSM980 Airyscan 8Y using Plan Apo Oil 1.4 numerical aperture 63x objective. TDP1-mCherry was photo-bleached by the Zen image analysis module of Zeiss Zen Blue software ver 3.5. A 561-nm laser was used to photobleach TDP1-mCherry inclusions. The region corresponding to the TDP1-mCherry inclusion was photo-bleached with maximal 561-nm laser power to obtain more than 80% of photo-bleaching and recovery with 1% of laser power. Spot bleach was performed for three iterations. Fluorescence intensities were measured by acquisitions in prebleached, bleached areas every 2 s 26 times during the post-bleached recovery. Intensity data of bleach and nonbleached inclusions (mean intensity) were extracted from the Zen Blue software and analyzed with Excel to calculate fluorescent protein mobility. The fraction of the fluorescent protein that was mobile was calculated by comparing the fluorescence intensities of nonbleached and bleached TDP1-mCherry inclusions. The mean prebleach value represents the maximum intensity to which the bleached region could possibly recovery. Representative images are presented following the max-project of Z-stacked images using the maximum intensity projection type with the Fiji ImageJ software (version 2.3.0/1.53f).

### FRAP in cells

FRAP measurements in U2OS, SH-SY5Y, and iPS cells were performed on a Leica SP8 confocal microscope equipped with a 405-nm and white light lasers using a 63× oil immersion objective. Bleaching and recovery conditions were as follows: 10 s prebleaching recording, 1 s bleach with a laser intensity of 100% at 405 nm, followed by 120 s recovery (130 time points). Analysis of the recovery curves was carried out using a custom-written FIJI/ImageJ routine. The equation used for FRAP analysis is as follows: {(*I*_bleach_ − *I*_background_)/[*I*_bleach_(*t*_0_) − *I*_background_(*t*_0_)]}/{(*I*_total_ − *I*_background_)/[*I*_total_(*t*_0_) − *I*_background_(*t*_0_)]}, where *I*_total_ is the fluorescence intensity of the entire cellular structure, *I*_bleach_ represents the fluorescence intensity in the bleach area, and *I*_background_ is the background of the camera offset. Fluorescent density analysis was performed using FIJI/ImageJ and selecting the specific region of interest (ROI). When necessary, image drift correction was applied using the StackReg plug-in function of the FIJI software suite before FRAP analysis. FRAP curves were averaged to obtain the mean and standard deviation.

### RT-qPCR

Total RNA was isolated from U2OS cells, iPSCs, and iPSC-MNs treated as indicated in the figure legends, using the ReliaPrep RNA Miniprep Systems (Promega) according to the manufacturer’s instruction. RNA (500 ng) was reverse transcribed using PrimeScript RT Master Mix (Takara), following the manufacturer’s instruction. PCR amplification was performed using TB Green Premix Ex Taq II (Tli RNase H Plus) (Takara). *RPL0* and *ATP5O* were used as reference genes for normalization of U2OS and iPS cells, respectively (*89*). Primers used for PCR analysis are listed in table S2.

### SUMO pathway protein purification protocols

The expression and purification of the SUMO pathway proteins was based on the protocols by Flotho *et al.* (*42*). The following Addgene plasmids were used: 53135 (pET28a-His-hAos1), 53136 (pET11d-hUba2), 53137 (pET23a-mUBC9), 53140 (pGEX-RanBP2 IR1 + M), and 53141 (pET28-HisYFP-Sp100). The bacteria strains used were *E. coli* BL21(DE3) RIL. Instead, pET11a-SUMO2 was expressed in *One Shot BL21 Star* (*DE3*) *Chemically Competent E. coli* (Thermo Fisher Scientific). All purification steps were carried out at 4°C, as previously described (*42*).

Recombinant GST-PIAS4 and TDP-43-GFP-MBP-6xHis were expressed in insect cells. GST-PIAS4–expressing cells were lysed in a buffer containing 40 mM Hepes, pH 7.5, 150 mM NaCl, and 2 mM EDTA, supplemented with fresh 1 mM DTT, 1 × EDTA-free protease inhibitor cocktail, and 100 μM Benzonase. The cleared lysate was applied to Glutathione Sepharose 4 FastFlow (Cytiva Life Science). GST-PIAS4 was eluted using a buffer containing 40 mM Hepes, pH 7.5, 150 mM NaCl, 10 mM KCl, 2 mM EDTA, 1 mM DTT, and 10 mM reduced glutathione, and incubated overnight with His-3C protease at 4°C to cleave off the GST tag. The protein was then separated from its cleaved tag via SEC using the HiLoad 16/60 Superdex 75 column (Cytiva Life Science).

TDP-43-GFP-MBP-6xHis expressing cells were lysed in a buffer containing 20 mM Hepes, pH 7.5, 150 mM NaCl, and 5% glycerol, supplemented with fresh 1 mM DTT. The cleared lysate was applied to HiTrap FF (Cytiva Life Science) to perform His-affinity chromatography, eluted in a buffer containing 250 mM imidazole, then subjected to MBP-affinity chromatography using MBPTrap FF (Cytiva Life Science). After elution with a buffer containing 20 mM maltose, the recombinant TDP-43-GFP-MBP-6xHis protein was loaded onto HiPrep 25/10 desalting column for buffer exchange with the SEC buffer (20 mM Hepes pH 7.5, 1 M NaCl, 5% glycerol, 1 mM DTT, and 1 mM EDTA). The eluted protein was then incubated overnight with His-3C protease at 4°C to cleave off the tags and purified via SEC using HiLoad 26/60 Superdex 200 pg (Cytiva Life Science). All purified proteins were aliquoted, flash frozen in liquid nitrogen, and stored at −80°C.

### In vitro SUMOylation assays

All in vitro SUMOylation assays were performed in the SUMOylation assay buffer (SAB), which contained 20 mM Hepes, pH 7.3, 110 mM KOAc, 2 mM Mg(OAc)2, ovalbumin (0.2 mg/ml), and 1 mM DTT. All samples were prepared on ice by mixing together 165 nM AOS/UBA2, 50 nM Ubc9, 100 nM E3 (GST-RanBP2 or PIAS4), 200 nM substrate (TDP-43-GFP, YFP-Sp100, or NTD-GFP), 10 mΜ SUMO2, and 10 mM ATP, in a final reaction volume of 20 μl. The reaction was carried out for 2 hours at 37°C, then stopped by the addition of 4× SDS loading buffer. The samples were analyzed via SDS-PAGE, using 10% acrylamide gels and directly imaged for the GFP fluorescence of the substrate using Amersham Typhoon Biomolecular Imager (Cytiva). Quantification of the modified substrate was obtained using the ImageQuant analysis software. When the assay was carried out in the presence of UG-rich RNA (UG_35_), TDP-43-GFP and the RNA were allowed to bind in the buffer for 5 min before the addition of SUMO2 and the SUMO enzymes.

### Add-back splicing assay

HeLa cells were transfected with two rounds of siRNAs specific for Firefly luciferase (negative control) or TDP-43, as previously published (*39*). On day 2, cells were transfected with the pTB-CFTR_Ex9 C155T plasmid alone or with either siRNA-resistant TDP-43 WT (pCMV4flagsiRTDP-43 WT) or TDP-43 K/R variants (pCMV4flagsiR TDP-43 N 3K/R, RRM1 5K/R, RRM1 10K/R, C 2K/R and N-C 5K/R) using Effectene (Qiagen) and following the manufacturer’s instructions. RNA extraction was performed using the RNeasy kit (Qiagen), followed by PCR using Taq Biolabs (New England Biolabs). The PCR conditions were as follows: 35 amplification cycles (95°C for 45 s, 54°C for 45 s, and 72°C for 45 s), as previously published (*39*). The PCR products were then run and analyzed with QIAxcel High Resolution Kit (Qiagen). Inclusion levels of exons were quantified, followed by multiple *t* test analysis.

### Human postmortem tissue, immunofluorescence microscopy, and quantification

The postmortem tissue was obtained 6 to 30 hours after death. The demographic details of the patients with fALS, the patients with sALS, and the normal nonneurological controls included in this study are summarized in table S1. Human postmortem brain and spinal cord samples fixed in buffered formalin from patients with sALS (*n* = 8), patients with fALS TDP-43 (*n* = 2), patients with fALS C9orf72 (*n* = 5), and age-matched controls (*n* = 4) were obtained from the archives of the Department of Neuropathology, Amsterdam UMC, University of Amsterdam, and from the New York Brain Bank of Columbia University. All patients fulfilled the diagnostic criteria for ALS (El Escorial criteria) (*90*) as reviewed independently by several neuropathologists. Out of three, two of the TDP-43 fALS (see table S1) tissue samples were collected and banked at the New York Brain Bank of Columbia University with consent obtained from the patient’s next of kin, according to New York State law and the guidelines of the Department of Pathology of Columbia University and New York Presbyterian Hospital. The controls included in the present study were adult individuals without any history of neurological disease based on their last clinical evaluation.

Double immunofluorescence staining on human postmortem tissue was performed as described elsewhere (*91*, *92*). Briefly, deparaffinized tissue sections were heated in citrate buffer (pH 6) for 20 min in a pressure cooker for antigen retrieval. Sections were blocked with ready-to-use 10% normal goat serum for 1 hour at RT to avoid nonspecific binding, followed by incubation with primary antibodies at 4°C overnight. After a 10-min wash in TBS-T, the sections were incubated with an Alexa-conjugated secondary antibody (1:500 in TBS-T) at RT for 2 hours. Sections were rewashed in TBS-T (2× 10 min) and stained for 10 min with 0.1% Sudan Black in 80% ethanol to suppress endogenous lipofuscin autofluorescence. The sections were washed for 5 min in TBS-T and mounted with Vectashield mounting medium (Vector Laboratories) containing DAPI.

To assess the number of MNs and the presence or absence of TDP-43 aggregates, as well as to quantify PIAS4 immunoreactivity, only large MNs with clear morphology and visible nuclei were manually counted using a 20× and 40× objective lens. We analyzed three sections from each of the following groups: patients with sALS (*n* = 8), patients with fALS TDP-43 (*n* = 2), patients with fALS C9orf72 (*n* = 5), and age-matched controls (*n* = 4). Quantification of PIAS4 immunoreactivity within the MNs was conducted on randomly selected images of the ventral horn of the lumbar spinal cord. Multiple images were captured to ensure that all relevant MNs were included. Several ROIs were defined, independent of the area of suppressed lipofuscin autofluorescence within each MN. ROI area was kept constant throughout the analysis. The analysis criteria also considered the immunoreactivity of PIAS4 in the presence or absence of TDP-43 aggregates.

### Ethics statement

All procedures involving the use of postmortem tissue samples were performed under the institutional and national research committee’s ethical standards and with the 1964 Helsinki Declaration and its later amendments. The Ethical Committees of the Academic Medical Center, Amsterdam (W11 073) approved the studies. In the State of New York, research involving autopsy material does not meet the regulatory definition of “human subject research” and is not subject to institutional review board oversight.

### Quantification and statistical analysis

Statistical analyses on quantified imaging data were performed with one-way analysis of variance (ANOVA), followed by Student’s *t* test for comparisons between two groups or Bonferroni–Holm post hoc test for comparisons between three or more groups, using Daniel’s XL Toolbox or GraphPad Prism6 software. For quantification of PIAS4 immunoreactivity (see the above methods for details) in human autopsy tissue, we used one-way ANOVA, followed by Brown-Forsythe and Welch ANOVA tests: multiple comparisons using GraphPad Prism6 software. Where specified, multiple *t* test was used to compare multiple groups. Statistical details and exact *n* values for each experiment are reported in the figure legends.
