# Supplementary material for: SUMO2/3 conjugation of TDP-43 protects against aggregation
Source: Sci Adv. 2025 Feb 21;11(8):eadq2475. doi: 10.1126/sciadv.adq2475 (PMC11844728; doi:10.1126/sciadv.adq2475)
Supplement: Supplementary file 1 — Figs. S1 to S8 Tables S1 and S2 [file sciadv.adq2475_sm.pdf]

Science Advances
AAAS

Supplementary Materials for

SUMO2/3 conjugation of TDP-43 protects against aggregation

Enza Maria Verde *et al.*

Corresponding author: Serena Carra, serena.carra@unimore.it

Sci. Adv. **11**, eadq2475 (2025)
DOI: 10.1126/sciadv.adq2475

This PDF file includes:

Figs. S1 to S8
Tables S1 and S2

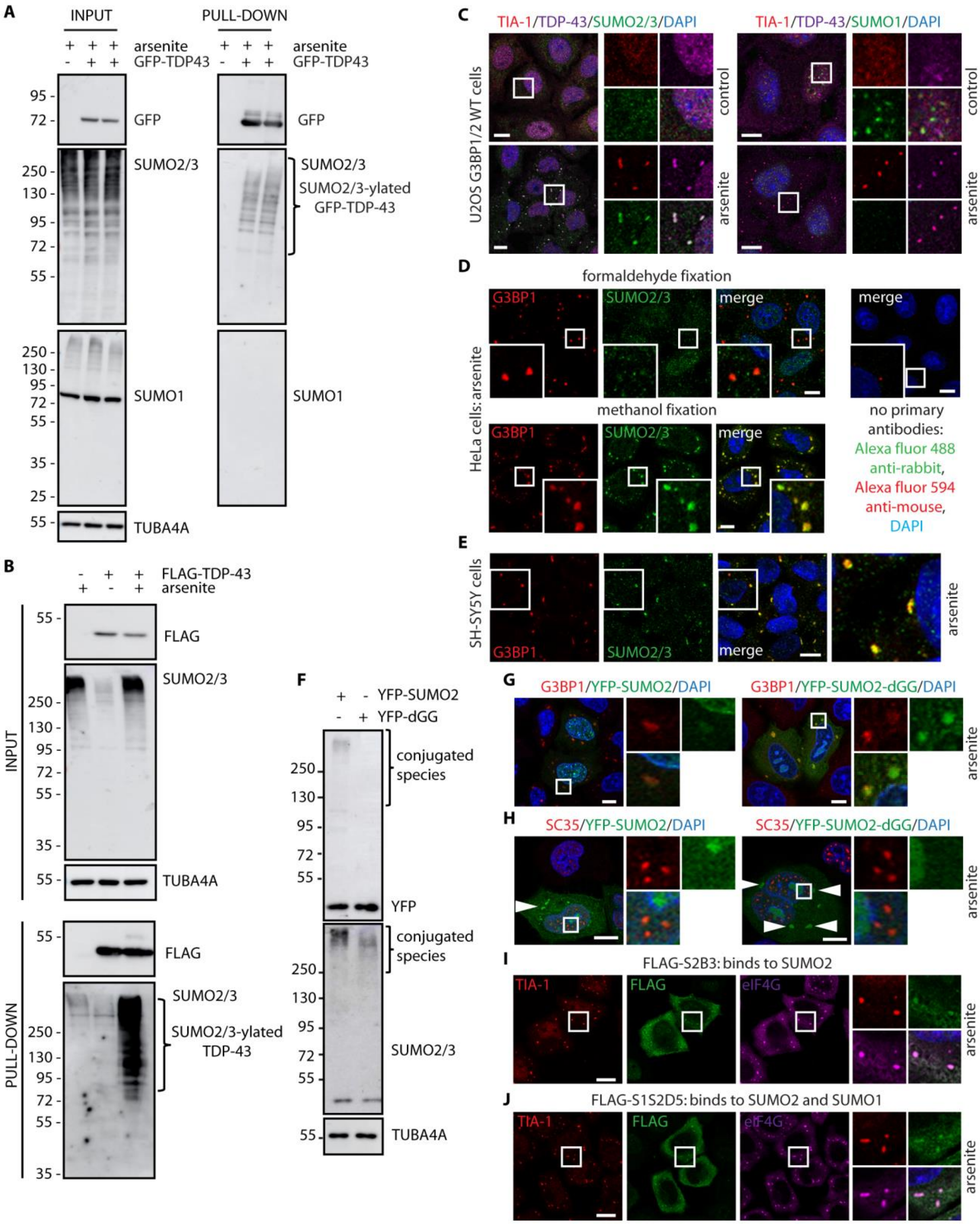
A
INPUT
PULL-DOWN
arsenite
GFP-TDP43
95
72
GFP
SUMO2/3
250
130
95
72
55
SUMO2/3-ylated GFP-TDP-43
SUMO1
35
25
TUBA4A
B
FLAG-TDP-43
arsenite
INPUT
PULL-DOWN
FLAG
SUMO2/3-ylated TDP-43
C
TIA-1/TDP-43/SUMO2/3/DAPI
TIA-1/TDP-43/SUMO1/DAPI
U2OS G3BP1/2 WT cells
control
arsenite
D
formaldehyde fixation
methanol fixation
HeLa cells: arsenite
G3BP1
SUMO2/3
merge
no primary antibodies:
Alexa fluor 488 anti-rabbit,
Alexa fluor 594 anti-mouse,
DAPI
E
SH-SY5Y cells
F
YFP-SUMO2
YFP-dGG
conjugated species
YFP
G
G3BP1/YFP-SUMO2/DAPI
G3BP1/YFP-SUMO2-dGG/DAPI
H
SC35/YFP-SUMO2/DAPI
SC35/YFP-SUMO2-dGG/DAPI
I
FLAG-S2B3: binds to SUMO2
TIA-1
FLAG
eIF4G
J
FLAG-S1S2D5: binds to SUMO2 and SUMO1

Fig. S1. Conjugated and free SUMO2/3 colocalizes with TDP-43 inside SGs. Related to Fig. 1.

(A) Immunoprecipitation under denaturing conditions of GFP-TDP-43 from U2OS cells treated with arsenite (500 µM, 1 hr). Lane 1 corresponds to the negative control (cells not expressing GFP-TDP-43), while lanes 2 and 3 correspond to two independent samples expressing GFP-TDP-43. Immunoblots of denatured total protein (input) and beads (pull-down) fractions with antibodies specific for SUMO1 and SUMO2/3 are shown. TUBA4A was used as loading control. The SUMO1 band migrating between 72 and 95 kDa in the input fraction corresponds to SUMO1-RanGAP.

(B) Immunoprecipitation under denaturing conditions of FLAG-TDP-43 from U2OS cells untreated or treated with arsenite (500 µM, 1 hr). Immunoblots of denatured total protein (input) and beads (pull-down) fractions are shown. TUBA4A was used as loading control.

(C) Confocal imaging of TIA-1, TDP-43, SUMO1, SUMO2/3 and DAPI in untreated and arsenite-treated U2OS cells. Scale bar, 10 µm.

(D) Confocal imaging of G3BP1, SUMO2/3 and DAPI in arsenite-treated HeLa cells fixed with formaldehyde or methanol. Scale bar, 10 µm.

(E) Confocal imaging of G3BP1, SUMO2/3 and DAPI in asenite-treated SH-SY5Y cells. Scale bar, 10 µm.

(F) Immunoblots of HeLa Kyoto cells overexpressing YFP-SUMO2 or dGG.

(G, H) Confocal imaging of YFP-SUMO2, dGG, DAPI and either G3BP1 (G) or SC35 (H) in arsenite-treated HeLa Kyoto cells. Scale bar, 10 µm.

(I, J) Confocal imaging of FLAG-affimers (S2B3, I or S1S2D5, J), TIA-1, eIF4G and DAPI in arsenite-treated HeLa Kyoto cells. Scale bar, 10 µm.

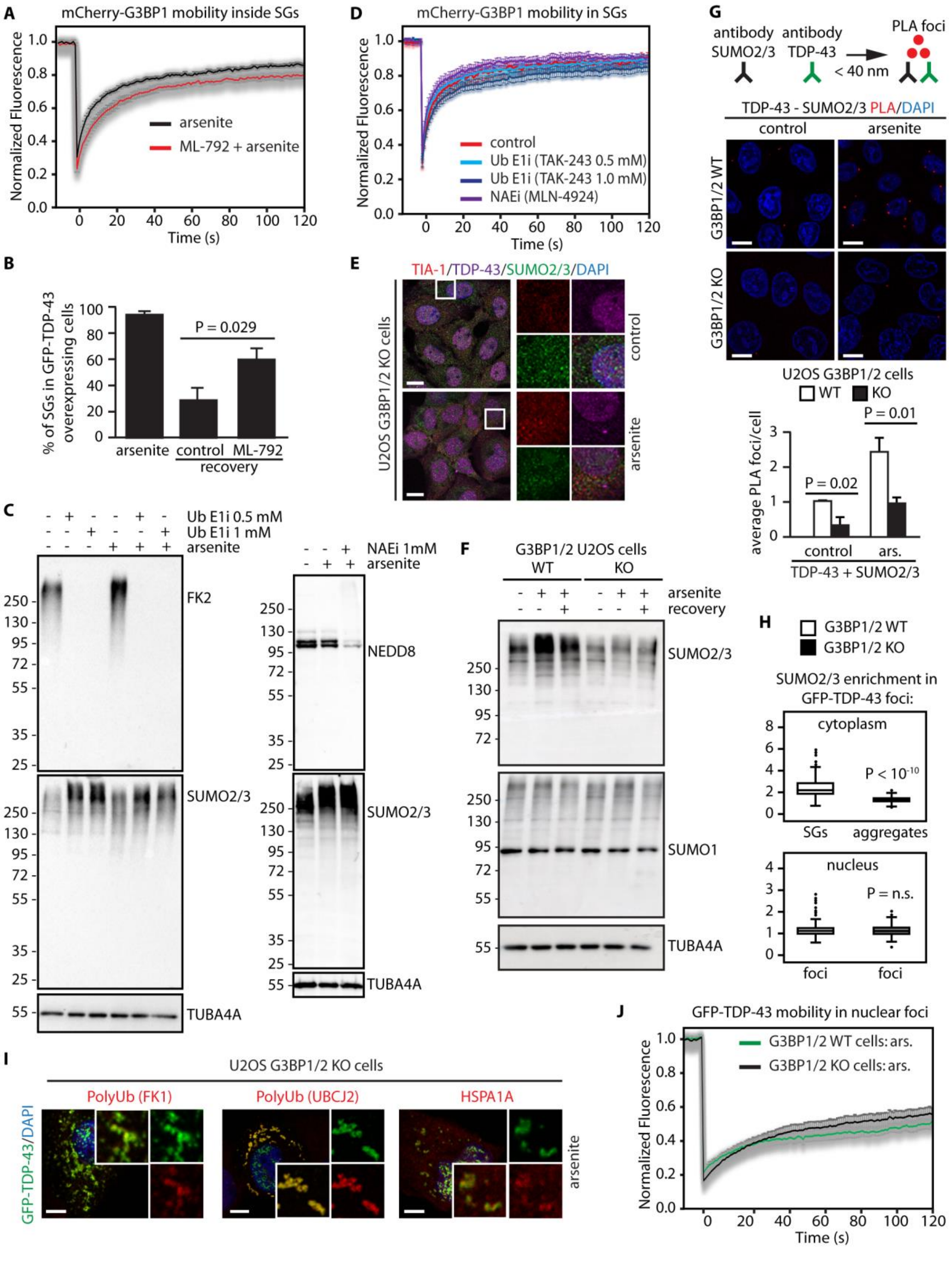
A
mCherry-G3BP1 mobility inside SGs
Normalized Fluorescence
Time (s)
arsenite
ML-792 + arsenite
B
% of SGs in GFP-TDP-43 overexpressing cells
P = 0.029
arsenite
control
ML-792
recovery
C
Ub E1i 0.5 mM
Ub E1i 1 mM
arsenite
FK2
SUMO2/3
TUBA4A
NAEi 1mM
NEDD8
D
mCherry-G3BP1 mobility in SGs
control
Ub E1i (TAK-243 0.5 mM)
Ub E1i (TAK-243 1.0 mM)
NAEi (MLN-4924)
E
TIA-1/TDP-43/SUMO2/3/DAPI
U2OS G3BP1/2 KO cells
control
arsenite
F
G3BP1/2 U2OS cells
WT
KO
recovery
SUMO1
G
antibody SUMO2/3
antibody TDP-43
PLA foci
< 40 nm
TDP-43 - SUMO2/3 PLA/DAPI
G3BP1/2 WT
G3BP1/2 KO
U2OS G3BP1/2 cells
WT
KO
average PLA foci/cell
P = 0.02
P = 0.01
ars.
TDP-43 + SUMO2/3
H
G3BP1/2 WT
G3BP1/2 KO
SUMO2/3 enrichment in GFP-TDP-43 foci:
cytoplasm
P < 10-10
SGs
aggregates
nucleus
P = n.s.
foci
I
U2OS G3BP1/2 KO cells
PolyUb (FK1)
PolyUb (UBCJ2)
HSPA1A
GFP-TDP-43/DAPI
J
GFP-TDP-43 mobility in nuclear foci
G3BP1/2 WT cells: ars.
G3BP1/2 KO cells: ars.

Fig. S2. U2OS G3BP1/2 KO cells fail to induce protein SUMO2/3-ylation upon oxidative stress. Related to Fig. 2.

(A) mCherry-G3BP1 FRAP curves inside SGs in U2OS cells treated with arsenite alone or with ML-792 (mean, SEM, n=11 and 13).

(B) Percentage of GFP-TDP-43 overexpressing U2OS cells with SGs treated as indicated (mean, SEM, n=4. Cell number counted/condition: 343–593). One-way ANOVA, Bonferroni-Holm post-hoc test.

(C) Immunoblots of U2OS cells untreated or treated for 1 hr with ubiquitin (Ub E1i/TAK-243) or NEDD8 (NAEi/MLN-4924) activating enzymes' inhibitor prior to arsenite addition.

(D) mCherry-G3BP1 FRAP curves inside SGs in U2OS cells during arsenite stress with a pre-treatment with Ub E1i or NAEi1 (mean, SEM, n=3, control; n=9, Ub E1i 0.5 μM; n=8, Ub E1i 1.0 μM; n=5, NAEi).

(E) Endogenous TIA-1, TDP-43, SUMO2/3 and DAPI confocal imaging in control and arsenite-treated U2OS G3BP1/2 KO cells. Scale bar, 10 μm.

(F) Immunoblots of U2OS G3BP1/2 WT and KO cells untreated or arsenite-treated, followed by 1 hr recovery in drug-free medium.

(G) Schematic representation of PLA with TDP-43 and SUMO2/3 antibodies. Representative confocal images and average PLA foci number/cell in U2OS G3BP1/2 WT and KO cells control or arsenite-treated (mean, SEM, n=3. Number of cells analyzed: 206-536/sample). One-way ANOVA, Bonferroni-Holm post-hoc test. Scale bar, 10 μm.

(H) SUMO2/3 enrichment inside GFP-TDP-43 cytoplasmic and nuclear foci in U2OS G3BP1/2 WT and KO cells arsenite-treated. Mean, SEM. Number of GFP-TDP-43 foci segmented: 238 SGs, WT cells; 222 cytoplasmic aggregates, KO cells; 318 and 210 nuclear foci in WT and KO cells. Student's t-test.

(I) Polyubiquitin (FK1 and UBCJ2 antibodies), HSPA1A and DAPI confocal imaging in arsenite-treated U2OS G3BP1/2 KO cells overexpressing GFP-TDP-43. Scale bar, 5 μm.

(J) GFP-TDP-43 FRAP curves inside nuclear foci in U2OS G3BP1/2 WT and KO cells arsenite-treated (mean, SEM, n=6 and 15).

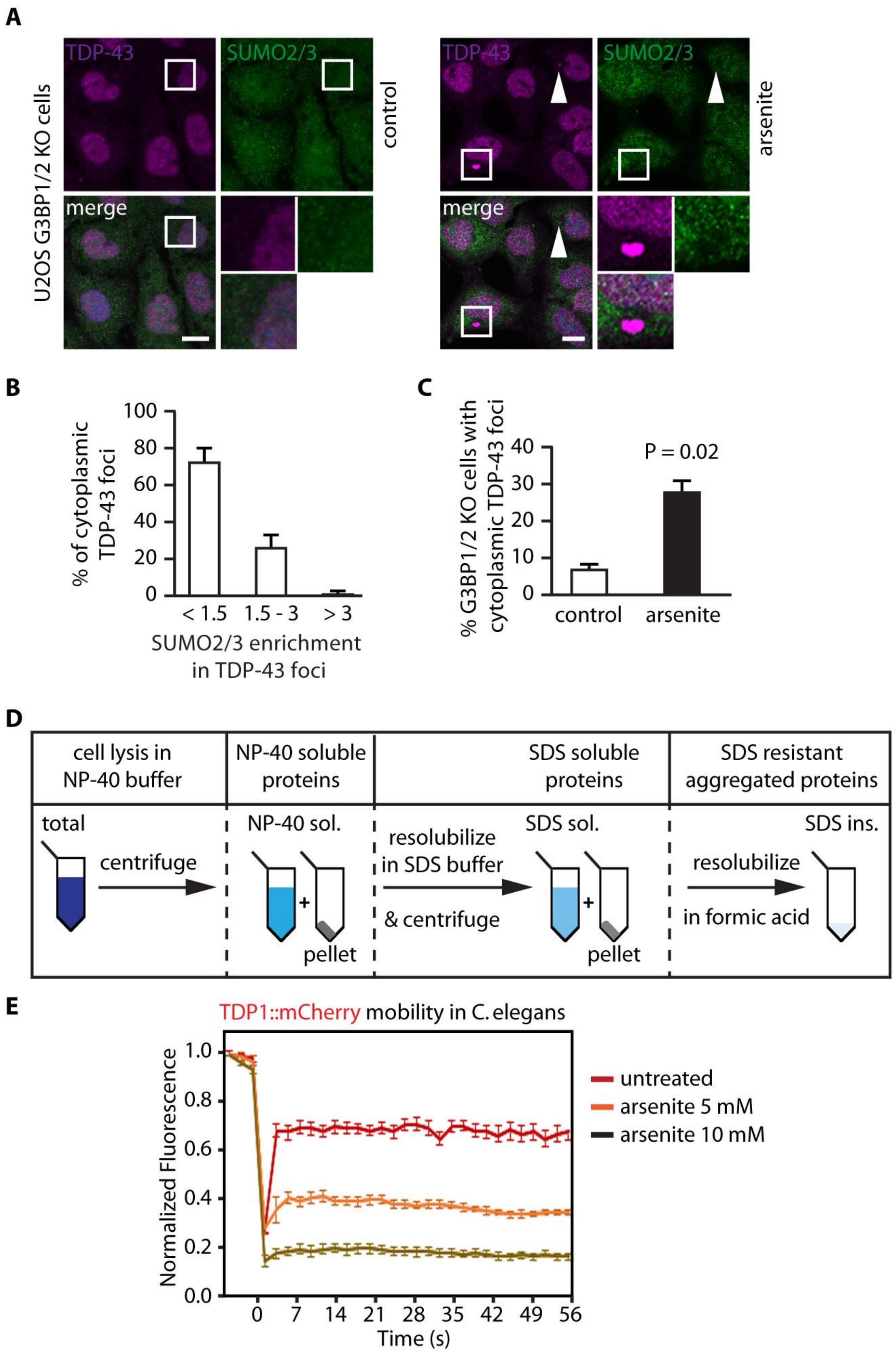

A
TDP-43
SUMO2/3
control
merge
U2OS G3BP1/2 KO cells
arsenite
B
% of cytoplasmic TDP-43 foci
100
80
60
40
20
0
< 1.5
1.5 - 3
> 3
SUMO2/3 enrichment in TDP-43 foci
C
% G3BP1/2 KO cells with cytoplasmic TDP-43 foci
P = 0.02
40
30
20
10
0
control
arsenite
D
cell lysis in NP-40 buffer
NP-40 soluble proteins
SDS soluble proteins
SDS resistant aggregated proteins
total
centrifuge
NP-40 sol.
pellet
resolubilize in SDS buffer
& centrifuge
SDS sol.
pellet
resolubilize
in formic acid
SDS ins.
E
TDP1::mCherry mobility in C. elegans
Normalized Fluorescence
1.0
0.8
0.6
0.4
0.2
0.0
0
7
14
21
28
35
42
49
56
Time (s)
untreated
arsenite 5 mM
arsenite 10 mM

Fig. S3. Oxidative stress decreases TDP1-mCherry mobility. Related to Fig. 3.

(A) Confocal imaging of endogenous TDP-43, SUMO2/3 and DAPI in untreated (control) and arsenite-treated U2OS G3BP1/2 KO cells. Selected region and white arrowhead indicate TDP-43 positive cytoplasmic foci. Scale bar, 10 µm.

(B) Quantification of the enrichment of SUMO2/3 inside TDP-43 cytoplasmic foci in arsenite-treated U2OS G3BP1/2 KO cells (mean, SEM, n=3; number of TDP-43 positive foci quantified in each sample: 188; 208; 294). One-way ANOVA, followed by Bonferroni-Holm post-hoc test.

(C) Quantification of the percentage of untreated (control) and arsenite-treated U2OS G3BP1/2 KO cells with TDP-43 positive cytoplasmic foci (mean, SEM; control, n=3). Student's t-test.

(D) Schematic representation of the method employed for the sequential fractionation of NP-40 soluble, SDS soluble and insoluble proteins from U2OS cells untreated or treated with arsenite with or without a pre-treatment with ML-792.

(E) FRAP curves of TDP1-mCherry in cells of living worms untreated or exposed to 5 and 10 mM arsenite, respectively (mean, SEM, n=8-10).

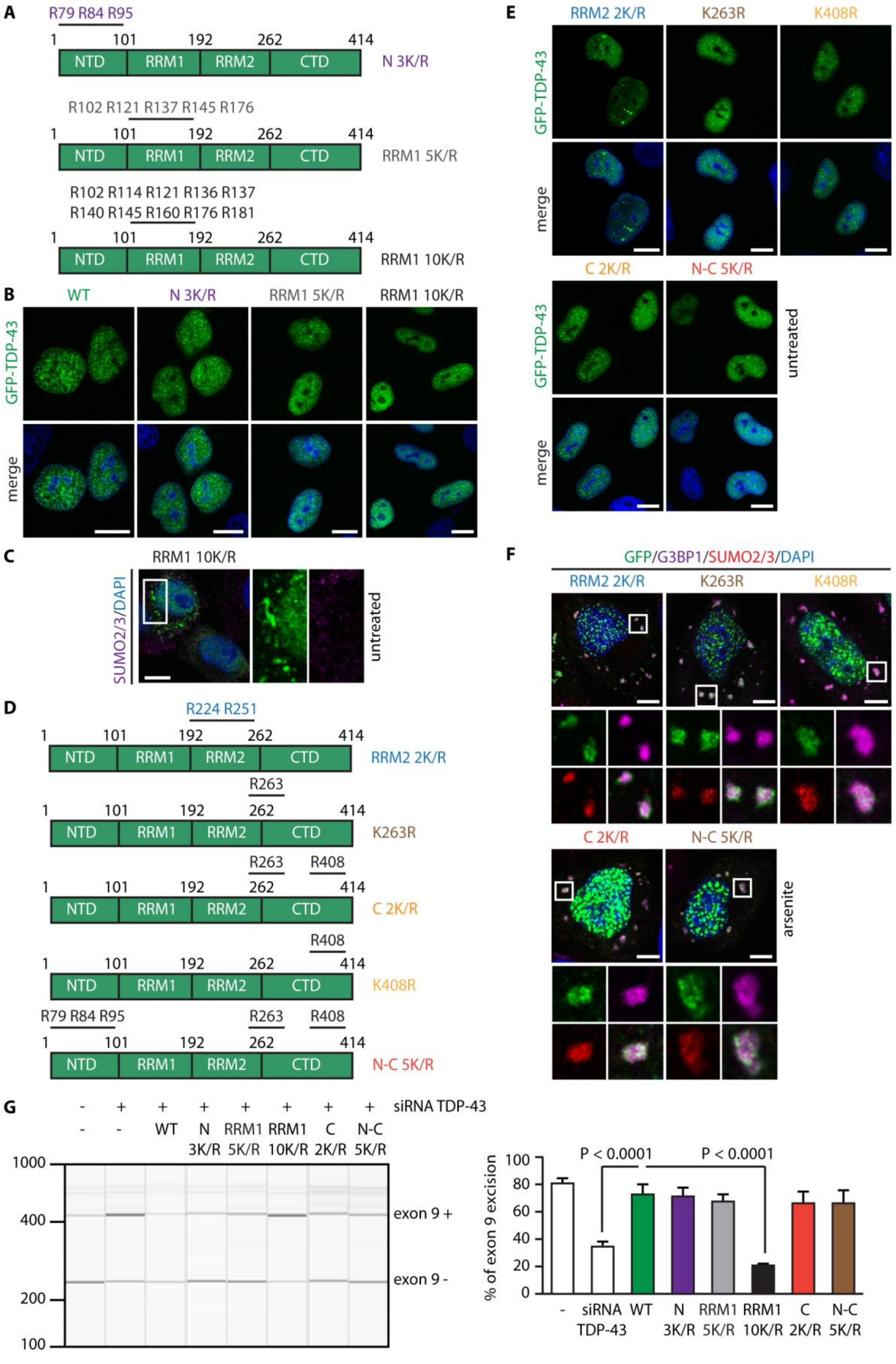
A
R79 R84 R95
1 101 192 262 414
NTD RRM1 RRM2 CTD
N 3K/R
R102 R121 R137 R145 R176
RRM1 5K/R
R102 R114 R121 R136 R137
R140 R145 R160 R176 R181
RRM1 10K/R
B
WT
N 3K/R
RRM1 5K/R
RRM1 10K/R
GFP-TDP-43
merge
C
RRM1 10K/R
SUMO2/3/DAPI
untreated
D
R224 R251
RRM2 2K/R
R263
K263R
R263 R408
C 2K/R
R408
K408R
R79 R84 R95 R263 R408
N-C 5K/R
E
RRM2 2K/R
K263R
K408R
GFP-TDP-43
merge
untreated
C 2K/R
N-C 5K/R
F
GFP/G3BP1/SUMO2/3/DAPI
arsenite
G
siRNA TDP-43
WT
N 3K/R
RRM1 5K/R
RRM1 10K/R
C 2K/R
N-C 5K/R
1000
400
200
100
exon 9 +
exon 9 -
P < 0.0001
P < 0.0001
% of exon 9 excision
100
80
60
40
20
0
-
siRNA TDP-43

Fig. S4. Subcellular distribution of GFP-TDP-43 K/R variants. Related to Fig. 4.

(A) Schematic representation of the GFP-TDP-43 N 3K/R, RRM1 5 and 10 K/R variants generated in this study.

(B) Confocal imaging of GFP-TDP-43 WT and the N 3K/R, RRM1 5 and 10 K/R variants overexpressed in U2OS cells for 24 hrs. DAPI is shown. Scale bar, 10 μm.

(C) Confocal imaging of SUMO2/3 and DAPI in U2OS cells overexpressing GFP-TDP-43 RRM1 10K/R and showing the formation of cytoplasmic aggregates that do not colocalize with SUMO2/3. Scale bar, 10 μm.

(D) Schematic representation of the GFP-TDP-43 RRM2 2K/R, C- and N-terminus variants generated in this study.

(E) Confocal imaging of GFP-TDP-43 WT and the RRM2 2K/R, C- and N-terminus variants overexpressed in U2OS cells for 24 hrs. DAPI is shown. Scale bar, 10 μm.

(F) Confocal imaging of GFP-TDP-43, G3BP1, SUMO2/3 and DAPI in U2OS cells transfected as described in E and exposed to sodium arsenite (500 μM, 1 hr). Scale bar, 5 μm.

(G) CFTR exon 9 minigene (C155T) analysis. Representative images and quantification of RT-PCR of CFTR exon 9 isoforms in cells expressing endogenous TDP-43 (-) or transfected with a siRNA specific for TDP-43, followed by subsequent overexpression (add back) of either siRNA-resistant TDP-43 WT, and the indicated K/R variants. The % of CFTR exon 9 excision is shown (mean, SEM, n = 3). $P < 0.0001$, multiple t-test.

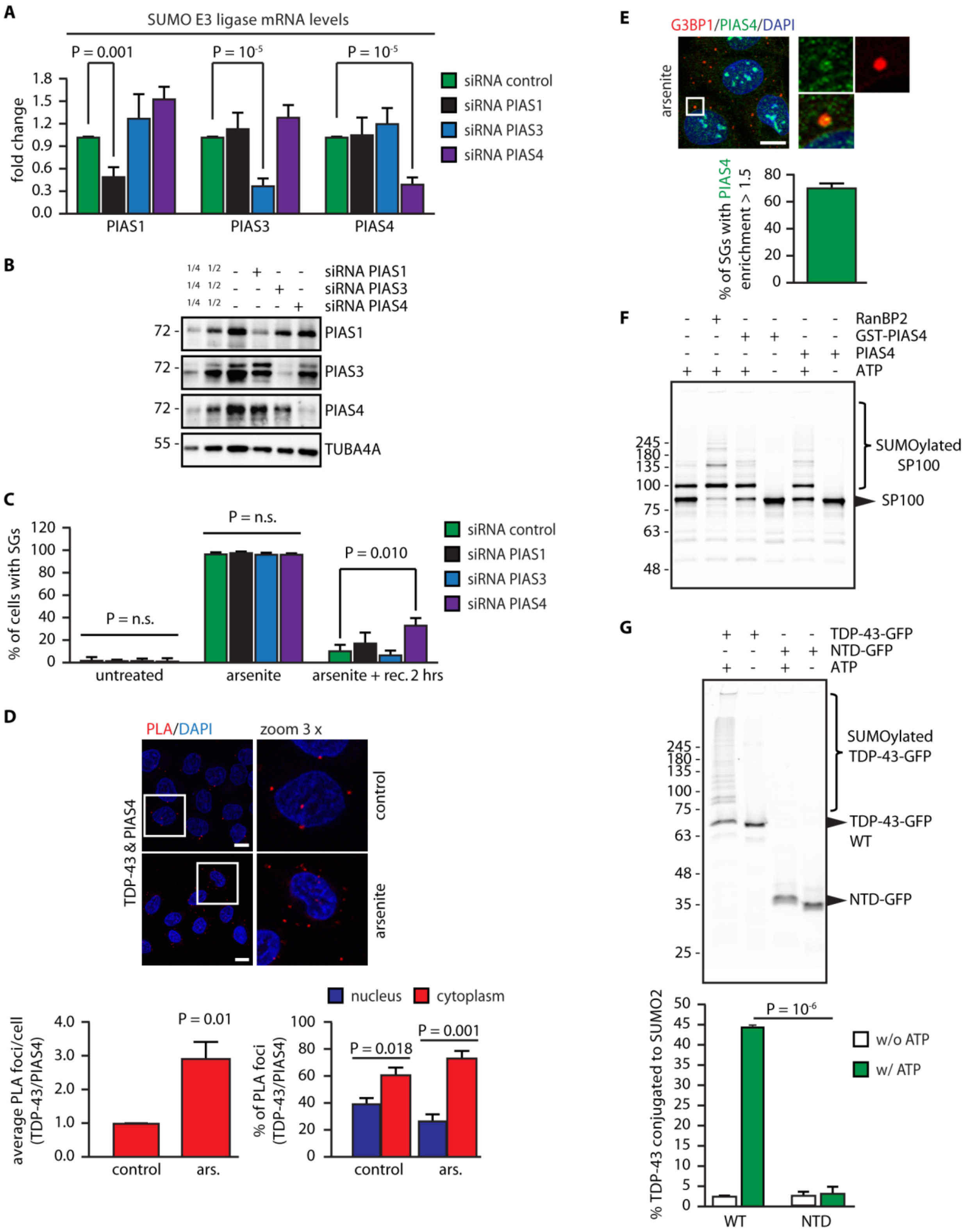

A
SUMO E3 ligase mRNA levels
P = 0.001
P = 10-5
P = 10-5
fold change
siRNA control
siRNA PIAS1
siRNA PIAS3
siRNA PIAS4
PIAS1
PIAS3
PIAS4
B
siRNA PIAS1
siRNA PIAS3
siRNA PIAS4
PIAS1
PIAS3
PIAS4
TUBA4A
C
% of cells with SGs
P = n.s.
P = n.s.
P = 0.010
untreated
arsenite
arsenite + rec. 2 hrs
D
PLA/DAPI
zoom 3 x
TDP-43 & PIAS4
control
arsenite
nucleus
cytoplasm
average PLA foci/cell (TDP-43/PIAS4)
P = 0.01
control
ars.
% of PLA foci (TDP-43/PIAS4)
P = 0.018
P = 0.001
E
G3BP1/PIAS4/DAPI
arsenite
% of SGs with PIAS4 enrichment > 1.5
F
RanBP2
GST-PIAS4
PIAS4
ATP
SUMOylated SP100
SP100
G
TDP-43-GFP
NTD-GFP
ATP
SUMOylated TDP-43-GFP
TDP-43-GFP WT
NTD-GFP
% TDP-43 conjugated to SUMO2
P = 10-6
w/o ATP
w/ ATP
WT
NTD

Fig. S5. TDP-43/PIAS4 proximity increased upon arsenite treatment. Related to Fig. 5.

(A) qPCR analysis of PIAS1, PIAS3 and PIAS4 mRNA levels in U2OS cells lipofected for 72 hrs with either a control non-targeting siRNA or with siRNA specific for PIAS1, PIAS3 and PIAS4 (mean, SEM, n=4). One-way ANOVA, followed by Bonferroni-Holm post-hoc test.

(B) Immunoblots of endogenous PIAS1, PIAS3 and PIAS4 in U2OS cells lipofected as reported in A.

(C) Percentage of cells bearing SGs in U2OS cells lipofected as reported in A and left untreated or treated with arsenite (500 μM for 45 min); where indicated cells were allowed to recover in drug-free medium for 2 hrs after arsenite treatment (mean, SEM, n=3). One-way ANOVA, followed by Bonferroni-Holm post-hoc test.

(D) Representative images and percentage of PLA foci/cell in U2OS cells untreated or arsenite-treated (500 μM, 45 min) and incubated with TDP-43 and PIAS4 antibodies; the percentage of TDP-43/PIAS4 PLA foci localized in the nucleus and in the cytoplasm is also shown (mean, SEM, n=3. Total number of cells analyzed: 368-1052/sample). One-way ANOVA, followed by Bonferroni-Holm post-hoc test. Scale bar, 10 μm.

(E) Confocal imaging of PIAS4 and G3BP1 in arsenite-treated U2OS cells. Quantification of the percentage of SGs with a PIAS4 enrichment above 1.5 (mean, SEM, n=13557 automatically segmented SGs). Scale bar, 10 μm.

(F) *In vitro* SUMO2-ylation of YFP-Sp100 in absence or presence of either RanBP2, GST-PIAS4, untagged PIAS4 and ATP.

(G) *In vitro* SUMO2-ylation of TDP-43-GFP and a truncated version of TDP-43 consisting of the N-terminal domain (NTD) fused to GFP. TDP-43-GFP and NTD-GFP were incubated with recombinant PIAS4, in absence or presence of ATP; representative image and quantification are shown (mean, SEM, n=3). One-way ANOVA, followed by Bonferroni-Holm post-hoc test.

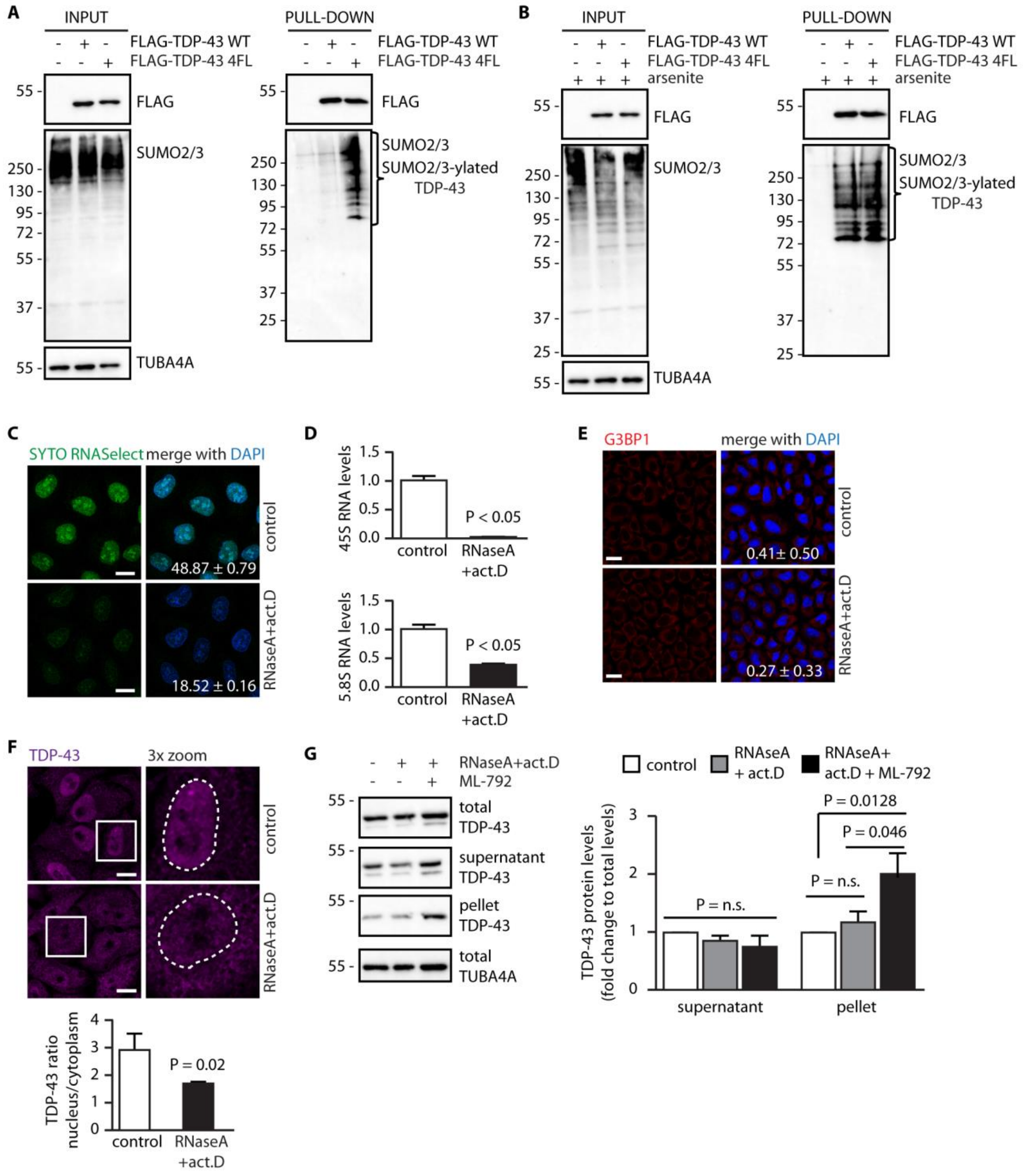

Fig. S6. RNA degradation and transcription inhibition induce the mislocalization of TDP-43 into the cytoplasm. Related to Fig. 6.

(A, B) Immunoprecipitation under denaturing conditions of FLAG-TDP-43 WT and 4FL from His10-SUMO2 U2OS cells untreated (A) or treated with arsenite (500 μM, 1 hr, B). Immunoblots

of denatured total protein (input) and beads (pull-down) fractions are shown. TUBA4A was used as loading control.
(C) Confocal imaging of SYTO RNASelect in U2OS cells untreated or treated with RNAse A (10 μM) and actinomycin D (4 μM) for 2 hrs (mean, SEM, n=423, control; n=558, RNAseA+act.D). Scale bar, 10 μm.
(D) qPCR analysis of 45S and 5.8S rRNA in U2OS cells treated as described in A (mean, SEM, n=3). Student's t-test.
(E) Confocal imaging of G3BP1 in U2OS cells treated as described in C; the % of SG-positive cells is shown (mean, SEM, n=3. Total number of cells counted: 401, control; 361, RNAseA+act.D). Scale bar, 20 μm.
(F) Confocal imaging of TDP-43 in U2OS cells treated as described in C. Quantification of TDP-43 nucleus/cytoplasm ratio is shown (mean, SEM, n=3. Total number of cells analyzed: 606, control; 642, RNAseA+act.D). Student's t-test. Scale bar, 10 μm.
(G) Fractionation of soluble (supernatant) and insoluble (pellet) proteins from U2OS cells left untreated or treated with RNAse A (10 μM) and actinomycin D (4 μM); where indicate cells were pre-treated with ML-792 (2 μM) for 2 hrs. Immunoblots of denatured total proteins and fractions are shown. TUBA4A was used as loading control. Quantification of endogenous TDP-43 levels in the supernatant and pellet fractions, expressed as fold-change compared to the control condition (mean, SEM, n=4). TUBA4A was used as loading control. One-way ANOVA, followed by Bonferroni-Holm post-hoc test.

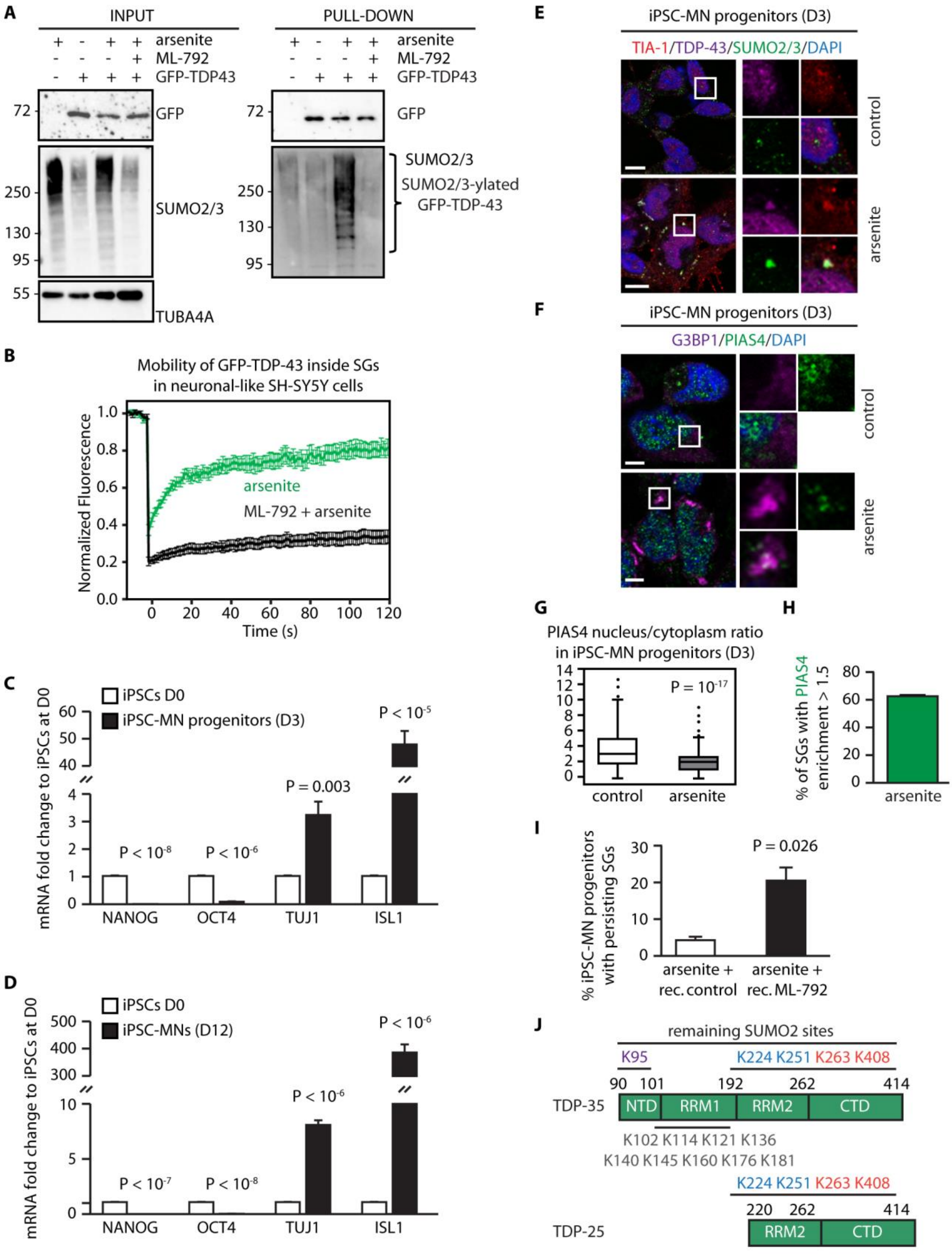
A
INPUT
PULL-DOWN
arsenite
ML-792
GFP-TDP43
72
GFP
250
130
95
SUMO2/3
55
TUBA4A
SUMO2/3
SUMO2/3-ylated GFP-TDP-43
B
Mobility of GFP-TDP-43 inside SGs in neuronal-like SH-SY5Y cells
Normalized Fluorescence
Time (s)
arsenite
ML-792 + arsenite
C
iPSCs D0
iPSC-MN progenitors (D3)
mRNA fold change to iPSCs at D0
P < 10-8
P < 10-6
P = 0.003
P < 10-5
NANOG
OCT4
TUJ1
ISL1
D
iPSCs D0
iPSC-MNs (D12)
mRNA fold change to iPSCs at D0
P < 10-7
P < 10-8
P < 10-6
P < 10-6
NANOG
OCT4
TUJ1
ISL1
E
iPSC-MN progenitors (D3)
TIA-1/TDP-43/SUMO2/3/DAPI
control
arsenite
F
iPSC-MN progenitors (D3)
G3BP1/PIAS4/DAPI
control
arsenite
G
PIAS4 nucleus/cytoplasm ratio in iPSC-MN progenitors (D3)
P = 10-17
control
arsenite
H
% of SGs with PIAS4 enrichment > 1.5
arsenite
I
% iPSC-MN progenitors with persisting SGs
P = 0.026
arsenite + rec. control
arsenite + rec. ML-792
J
remaining SUMO2 sites
K95
K224 K251 K263 K408
TDP-35
NTD
RRM1
RRM2
CTD
K102 K114 K121 K136
K140 K145 K160 K176 K181
K224 K251 K263 K408
TDP-25
RRM2
CTD

Fig. S7. SUMO2/3-ylation inhibition affects TDP-43 mobility inside SGs in SH-SY5Y cells. Related to Fig. 7.

(A) Immunoprecipitation under denaturing conditions of GFP-TDP-43 from SH-SY5Y cells untreated or arsenite-treated (500 μM, 1 hr); where indicated cells were incubated with ML-792 (2 μM, 2 hrs), prior to arsenite addition. Immunoblots of denatured total protein (input) and beads (pull-down) fractions are shown. TUBA4A was used as loading control.

(B) GFP-TDP-43 FRAP curves inside SGs in SH-SY5Y cells treated with arsenite alone or pre-treated with ML-792 (mean, SEM, n=9 and 12, respectively).

(C, D) qPCR analysis of NANOG, OCT4, TUJ1 and ISL1 mRNA in undifferentiated iPSCs (D0), iPSC-MN progenitors differentiated for 3 days (C; D3) or iPSC-MNs differentiated for 12 days (D; D12); (mean, SEM, n=3). Student’s t-test.

(E) Confocal imaging of endogenous TDP-43, SUMO2/3, TIA-1 and DAPI in iPSC-MN progenitors (D3) untreated or arsenite-treated (500 μM, 45 min). Scale bar, 10 μm.

(F) Confocal imaging of endogenous G3BP1, PIAS4 and DAPI in iPSC-MN progenitors differentiated (D3) untreated or arsenite-treated (500 μM, 45 min). Scale bar, 5 μm.

(G) Quantification of PIAS4 nucleus/cytoplasm ratio in iPSC-MN progenitors (D3) shown in panel F (mean, SEM; n=208, control; n=371, arsenite). Student’s t-test.

(H) Quantification of the percentage of SGs with a PIAS4 enrichment above 1.5 in iPSC-MN progenitors (D3) arsenite-treated and shown in panel F (mean, SEM, n=1532 segmented SGs).

(I) Quantification of the percentage of SG-positive iPSC-MN progenitors (D3) arsenite-treated followed by 2 hrs recovery in drug-free medium (rec. control) or co-treated with ML-792 (2 μM) and arsenite, followed by 2 hrs recovery with ML-792 (2 μM) (rec. ML-792). Mean, SEM, n=3. Student’s t-test.

(J) Schematic representation of the SUMO2-sites identified by mass spectrometry and retained in TDP-35 and TDP-25.

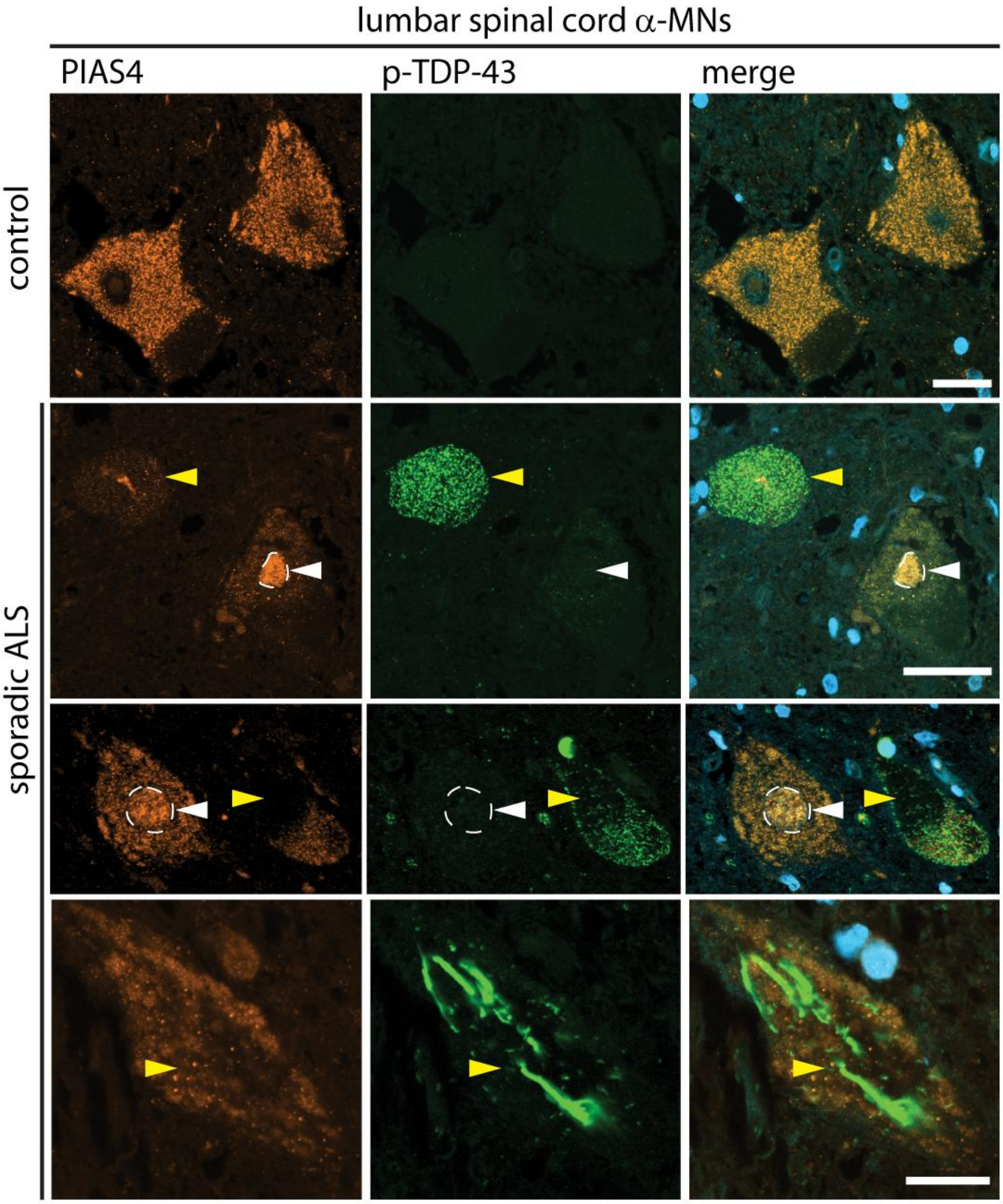

Fig. S8. Lumbar spinal cord α-MNs in sALS patients with TDP-43 pathology show reduced cytoplasmic PIAS4 immunoreactivity. Related to Fig. 8.

Lumbar spinal cord α-MNs from sALS cases showed a significant reduction in cytoplasmic PIAS4 immunoreactivity, accompanied by a strong increase in nuclear immunoreactivity (white

arrowheads) compared to control cases. MNs harboring pTDP-43 aggregates showed markedly reduced levels of PIAS4 (yellow arrowheads). Representative images from one of the 3 sections that were analyzed from sALS patients (n=8), and age-matched controls (n=4). Scale bars: 30 µm.

Table S1.

Patients examined in this study. PMI = Postmortem interval. fALS: familial ALS; sALS: sporadic ALS. Overall pTDP-43 load/Immunoreactivity in MNs: **+++:** (70-80 %) MNs showing TDP43 immunoreactivity, strong; **++:** 40-60% MNs showing TDP43 immunoreactivity, medium; **+:** below 30 % MNs showing TDP43 immunoreactivity, mild.

Case No.	Age	Gender	Cause of death	PMI (hrs)	Clinical diagnosis	Pathological Diagnosis	pTDP-43
1	65	M	respiratory	NA	fALS (c.1147A>G	TDP-43	+++
2	75	M	respiratory	10	fALS (c.881G>T	TDP-43	+++
1	68	M	euthanasia	12	fALS	C9orf72	++
2	69	M	muscle wasting	26	fALS	C9orf72	+++
3	61	M	unknown	24	fALS	C9orf72	+++
4	51	M	pneumonia	12	fALS	C9orf72	++
5	64	F	respiratory	24	fALS	C9orf72	+++
1	75	F	muscle wasting	36	sALS	sALS	+++
2	70	F	muscle wasting	24	sALS	sALS	++
3	64	M	urosepsis	24	sALS	sALS	+++
4	58	M	pneumonia	12	sALS	sALS	+++
5	65	F	respiratory	24	sALS	sALS	+++
6	76	F	muscle wasting	12	sALS	sALS	+++
7	68	F	pneumonia	24	sALS	sALS	+++
8	66	M	respiratory	<12	sALS	sALS	+++
1	54	M	sepsis	15	-	Normal	-
2	70	M	heart failure	7	-	Normal	-
3	81	M	organ failure	16	-	Normal	-
4	54	M	sepsis	15	-	Normal	-

Table S2.
Reagents and Tools used in this study.

Reagent/Resource	Reference or Source	Identifier or Catalog Number
**Antibodies**		
Donkey anti-Goat IgG (H+L), Alexa Fluor® 594	ThermoFisher	Cat#A-11058
Donkey anti-Goat IgG (H+L), Alexa Fluor® 647	ThermoFisher	Cat#A-21447
Donkey anti-Mouse IgG (H+L), Alexa Fluor® 488	ThermoFisher	Cat#A-21202
Donkey anti-Mouse IgG (H+L), Alexa Fluor® 594	ThermoFisher	Cat#A-21203
Donkey anti-Mouse IgG (H+L), Alexa Fluor® 647	ThermoFisher	Cat#A-31571
Donkey anti-Rabbit IgG (H+L), Alexa Fluor® 488	ThermoFisher	Cat#A-21206
Donkey anti-Rabbit IgG (H+L), Alexa Fluor® 594	ThermoFisher	Cat#A-21207
Donkey anti-Rabbit IgG (H+L), Alexa Fluor® 647	ThermoFisher	Cat#A-31573
eIF4G (H-300)	Santa Cruz Biotechnology	Cat#sc-11373
FLAG (M2)	Sigma-Aldrich	Cat#F1804
G3BP1	BD biosciences	Cat#611127
GADPH	Abcam	Cat#ab8245
GFP	Invitrogen	Cat#MA5-15256
HSPA1A	Stress Marq	Cat#SMC-100
Mouse IgG HRP Linked Whole Ab (from Sheep)	Cytiva	Cat#NA931V
NEDD8	Abcam	Cat#Ab81264
phospho Ser409/410 TDP-43	Cosmo bio	Cat#TIP-PTD-M01
PIAS1	Abcam	Cat#Ab109388
PIAS3	Cell Signaling	Cat#9042
PIAS4	Cell Signaling	Cat#4392
PIAS4 (PIASy)	GeneTex	Cat#GTX110497
Poly-Ubiquitin (FK1)	Enzo	Cat# BML-PW8805-0500
Poly-Ubiquitin (FK2)	Enzo	Cat#BML-PW8810-0100
Rabbit IgG HRP Linked Whole Ab (from Donkey)	Cytiva	Cat#NA934V
SC-35	Sigma-Aldrich	Cat#S4045
SUMO1	Abcam	Cat#Ab219724

SUMO2/3	Proteintech	Cat#10947-1-AP
SUMO2/3	Abcam	Cat#Ab 3742
TDP-43	Proteintech	Cat#60019-2-Ig
TDP-43	Proteintech	Cat#10782-2-AP
TIA-1	Santa Cruz Biotechnology	Cat#SC-1751
Tubulin	Sigma-Aldrich	Cat#T6074
UBCJ2	Enzo	Cat#Ab 5840-100
**Bacterial and virus strains**		
One Shot™ BL21 Star™ (DE3) Chemically Competent *E. coli*	ThermoFisher	Cat#601003
**Chemicals, Enzymes and other reagents**		
Actinomycin D	Sigma-Aldrich	Cat#A1410
ATP	Sigma-Aldrich	Cat#A2383
cOmplete Protease Inhibitor Cocktail	Roche	Cat#11836145001
cOmplete, EDTA-free Protease Inhibitor Cocktail	Roche	Cat#05056489001
DAPI	Sigma Aldrich	Cat#D9542
Duolink® in situ Detection Reagents Red	Sigma Aldrich	Cat#DUO92008
Duolink® in situ Mounting Media	Sigma Aldrich	Cat#DUO82040
Duolink® in situ PLA® probe Anti-Mouse MINUS	Sigma Aldrich	Cat#DUO92004
Duolink® in situ PLA® probe Anti-Rabbit PLUS	Sigma Aldrich	Cat#DUO92002
ML-792	MedChem Express	Cat#HY-108702
MLN4924	MedChem Express	Cat#HY-70062
N-etilmaleimide (NEM)	Sigma Aldrich	Cat#E3876
Normal Goat Serum (10%)	ThermoFisher	Cat#50062Z
ON-TARGETplus Human PIAS1 siRNA SMARTpool	Dharmacon	Cat#L008167000005
ON-TARGETplus Human PIAS3 siRNA SMARTpool	Dharmacon	Cat#L004164000005
ON-TARGETplus Human PIAS4 siRNA SMARTpool	Dharmacon	Cat#L006445000005
ON-TARGETplus Non-targeting siRNA#1	Dharmacon	Cat#D0018100105
PrimeScript RT Master Mix	Takara	Cat#RR036A
ReliaPrep™ RNA Miniprep Systems	Promega	Cat#Z6012
RNAseA	Roche	Cat#10109142001
Sodium arsenite	Carlo Erba	Cat# S7400
SYTO™ RNASelect™	Invitrogen	Cat#S32703
TAK-243 (MLN7243)	MedChem Express	Cat#HY-100487
Target Retrieval Solution, Citrate pH 6	Dako	Cat#S 2369
TB Green Premix Ex Taq	Takara	Cat#RR420A
TDP-43-GFP WT	This paper	N/A
TDP-43-GFP ΔRRM1-2	This paper	N/A

Vectashield Antifade mounting medium	Vector Laboratories	Cat#H-1000-10
**Experimental Models**		
Caenorhabditis elegans sup46::GFP; tdp1::mCherry	35	N/A
HeLa Kyoto cells	85	N/A
His-SUMO2-HEK-293T cells	This paper	N/A
iPSC line WTSli004-A with NIL	57	N/A
Sf9 cells	Expression Systems	Cat#94-001F
SH-SY5Y cells	ATCC	Cat#HTB-11
His10-SUMO2 U2OS cells	Dr. Alfred Vertegaal	N/A
U2OS cells	14	N/A
U2OS G3BP1/2 KO cells	14	N/A
**Oligonucleotides**		
5.8S-for and 5.8S-rev	89	5.8S-for (5'-ACTCGGCTCGTGCGTC-3'); 5.8S-rev (5'-GCGACGCTCAGACAGG-3')
ATP50	57	ATP5O-for and ATP5O-rev : ATP5O-for (5'-ACTCGGGTTTGACCTACAGC-3'); ATP5O-rev (5'-GGTACTGAAGCATCGCACCT-3')
cyclophilin-for and cyclophilin-rev	89	cyclophilin-for (5'-TGCCATCGCCAAGGAGTAG-3'); cyclophilin-rev (5'-TGCACAGACGGTCACTCAAA-3')
ILS1-for and ISL1-rev	57	ISL1-for (5'-AAGGTGGAGCTGCATTGGTTTG-3'); ISL1-rev (5'-TAAACCAGCTACAGGACAGGCC-3')
NANOG-for and NANOG-rev	57	NANOG-for (5'-CCAAATTCTCCTGCCAGTGAC-3'); NANOG-rev (5'-CACGTGGTTTCCAAACAAGAAA-3')
OCT4-for and OCT4-rev	57	OCT4-for (5'-ATGCATTCAAACTGA

		GGTGCCTGC-3'); OCT4-rev (5'-AACTTCACCTTCCCTCCAACCAGT-3')
PIAS1-for and PIAS1-rev	This paper	PIAS1-for (5’-GACCTCCTACACTCGTCTCG-3’); PIAS1-rev (5’-ACTGCCACTACTGCTTCCAT-3’)
PIAS3-for and PIAS3-rev	This paper	PIAS3-for (5’-TTGACTGCAGGAACCCTTCT-3’); PIAS3-rev (5’-AGACTTGTAGTGGCCACCTC-3’)
PIAS4-for and PIAS4-rev	This paper	PIAS4-for (5’-AGACCCTCAAGCCAGAAGTC-3’); PIAS4-rev (5’-GCTTCTCGTTGTTCTGTGGG-3’)
pre-rRNA 45S-for and pre-rRNA 45S-rev	89	pre-rRNA 45S-for (5’-GAACGGTGGTGTGTCGTTC-3’); pre-rRNA 45S-rev (5’-GCGTCTCGTCTCGTCTCACT-3’)
RPL0-for and RPL0-rev	89	RPL0-for (5'-TTAAACCCTGCGTGGCAATCC-3'); RPL0-rev (5'-CCACATTCCCCCGGATATGA-3')
TUJ1-for and TUJ1-rev	57	TUJ1-for (5'-CCCGGAACCATGGACAGTGT-3'); TUJ1-rev (5'-TGACCCTTGGCCCAGTTGTT-3')
**Recombinant DNA**		
FLAG-S1S2D5	26	N/A
FLAG-S2B3	26	N/A
FLAG-TDP-43	59	N/A
G3BP2-myc-DDK	Origene	Cat#RC203977
GFP-TDP-43 C 2K/R	This paper	N/A

GFP-TDP-43 K263R	This paper	N/A
GFP-TDP-43 K408R	This paper	N/A
GFP-TDP-43 N 3K/R	This paper	N/A
GFP-TDP-43 N-C 5K/R	This paper	N/A
GFP-TDP-43 RRM1 10K/R	This paper	N/A
GFP-TDP-43 RRM1 5K/R	This paper	N/A
GFP-TDP-43 RRM2 2K/R	This paper	N/A
GFP-TDP-43 WT	59	N/A
GFP-TDP-35	59	N/A
GFP-TDP-25	59	N/A
HIS-SUMO2	26	N/A
pET11a-SUMO2	Addgene	Cat#53142
pET11d-hUba2	Addgene	Cat#53136
pET23a-mUBC9	Addgene	Cat#53137
pET28a-His-hAos1	Addgene	Cat#53135
pET28-HisYFP-SP100	Addgene	Cat#53141
pGEX-RanBP2 IR1+M	Addgene	Cat#53140
pcDNA 3.1 mcherryG3BP1 (O-3507)	33	N/A
pCMV4flagsiR TDP-43 C 2K/R	This paper	N/A
pCMV4flagsiR TDP-43 N 3K/R	This paper	N/A
pCMV4flagsiR TDP-43 N-C 5K/R	This paper	N/A
pCMV4flagsiR TDP-43 RRM1 10K/R	This paper	N/A
pCMV4flagsiR TDP-43 RRM1 5K/R	This paper	N/A
pCMV4flagsiRTDP-43 WT	39	N/A
pOCC151-GST-PIAS4	This paper	N/A
pOCC180-pOEM1 TDP43-GFP-PS-MBP-HIS6	This paper	N/A
YFP-SUMO2	24	N/A
YFP-SUMO2-dGG	24	N/A
**Software and algorithms**		
Daniel's XL Toolbox	open-source add-in for Microsoft® Excel®	https://www.xltoolbox.net/
Fiji	NIH	https://fiji.sc/
Gel-Pro Analyzer 3.0	Informer Technologies	https://gel-pro-analyzer.software.informer.com/3.0/
GraphPad	GraphPad Prism for Windows	https://www.graphpad.com/
scanR V3.0	Olympus	https://www.olympus-lifescience.com/en/microscopes/inverted/scanr/
**Other**		
Amersham Protran Premium 0.45 NC nitrocellulose	Cytiva	Cat#10600003
Anti-FLAG M2 magnetic beads	Sigma Aldrich	Cat#M8832

GFP-TRAP BEADS	Chromoteck	Cat#gta-20
Lipofectamine 2000	ThermoFisher	Cat#11668019
Lipofectamine 3000	ThermoFisher	Cat#L3000015
Ni-NTA Magnetic Agarose Beads	QIAGEN	Cat#36113
WESTAR ETA C ULTRA 2.0	Cyanagen	Cat#XLS075,0100
WESTAR SUPERNOVA	Cyanagen	Cat#XLS3,0100
